# Jia-Ji Electro-Acupuncture Improves Locomotor Function With Spinal Cord Injury by Regulation of Autophagy Flux and Inhibition of Necroptosis

**DOI:** 10.3389/fnins.2020.616864

**Published:** 2021-01-22

**Authors:** Yin Hongna, Tian Hongzhao, Li Quan, Feng Delin, Liu Guijun, Lv Xiaolin, Guan Fulin, Sun Zhongren

**Affiliations:** ^1^Acupuncture Department, Heilongjiang University of Chinese Medicine, Harbin, China; ^2^Harbin Children’s Hospital, Harbin, China; ^3^Neurology Department, The First Affiliated Hospital of Harbin Medical University, Harbin, China

**Keywords:** Jia-Ji electro-acupuncture, spinal cord injury, motor function repair, autophagy flux, necroptosis, lysosome

## Abstract

Jia-Ji electro-acupuncture (EA) has been widely applied in clinic to exhibit curative effects on spinal cord injury (SCI). However, its underlying mechanisms leading to improvement of motor function after SCI remain unclear. Allen’s method was made by NYU Impactor M-III equipment to create the SCI rats model. Rats were randomly divided into four groups: Sham (only laminectomy), Model (SCI group), EA (SCI + Jia-Ji EA treatment), EA + CQ (SCI + Jia-Ji EA treatment + inhibitor chloroquine). Basso-Beattie-Bresnahan assessment showed improvement of hind limb motor function after Jia-Ji electro-acupuncture treatment. Histological change of injured spinal cord tissue was alleviated after treatment, observed by hematoxylin-eosin and Nissl staining. The mRNA and protein expression levels of RIPK1, RIPK3 and MLKL were decreased in EA group. Besides, the increased expression of LC3 and reduced expression of P62 after treatment compared with Model group, confirmed that Jia-Ji electro-acupuncture could enhance the autophagy flux. Electron microscopy imaging showed increasing the number of lysosomes, autophagosomes, and autolysosomes after Jia-Ji electro-acupuncture treatment. Furthermore, inhibition of lysosome function with CQ led to partly eliminate the effect of EA on reducing necroptosis. These data make the case that Jia-Ji electro-acupuncture treatment may improve locomotor function by promoting autophagy flux and inhibiting necroptosis.

## Introduction

Spinal cord injury (SCI) may cause structural and functional impairment of the spinal cord, leading to sensory, motor or autonomic nerve dysfunction, and ultimately affecting the patient’s physical, psychological, and social abilities (Singh et al., 2014). With its secondary complications in the long term, SCI is life-threatening and constitutes a considerable portion of the global social and economic burden (GBD 2016 Traumatic Brain Injury and Spinal Cord Injury Collaborators, 2019). The annual incidence of SCI in the United States is about 17,000 new cases per year (National Spinal Cord Injury Statistical Center, 2016). The global incidence of traumatic SCI ranges from 3.6 to 195.4 patients per million, and the numbers are still rising (Jazayeri et al., 2015).

The pathological mechanism of traumatic SCI involves primary injury (irreversible mechanical injury), and secondary injury (reversible) which is caused by a progressive cascade of tissue destruction and microenvironment changes, including oxidative stress, ischemia, inflammation, edema, glutamatergic excitotoxicity, ion imbalance, and apoptosis (Kumar et al., 2018). Rapid diagnosis and neuroprotective interventions at the stage of acute injury are therefore critical, and these treatments have the potential to significantly improve subsequent long-term functional recovery. Recent medical treatments attempt to reduce secondary damage and protect neurons that survive primary damage (Kwon et al., 2004). Some adjunctive therapies are used as an effective treatment of acute SCI clinically, such as corticosteroids (Schroeder et al., 2014), neuroprotectant agents (Grossman et al., 2014), and alternative therapy (electro-acupuncture) (Xiong et al., 2019).

As a Traditional Chinese medicine method, electro-acupuncture (EA) is a therapy in which a needle is inserted at an acupuncture point and attached to a trace amount of pulsed current to produce a synthetic electro-acupuncture stimulation. Previous studies have proved that the application of EA in the treatment of SCI is beneficial to the neurological and functional recovery of SCI (Ding et al., 2011; Huang et al., 2011). EA has been widely used in SCI therapy in the clinic to improve functional recovery after spinal cord and nerve muscle damage. However, its work mechanism is still unclear.

Inhibition of lysosomal/autophagy pathways and activation of necroptosis have been shown to lead to cell loss and tissue damage in some CNS trauma models (Sarkar et al., 2014; Lipinski et al., 2015). Necroptosis is proved to be associated with the pathogenesis of some neurodegenerative diseases, such as multiple sclerosis and amyotrophic lateral sclerosis (Ofengeim et al., 2015; Ito et al., 2016). It has been previously demonstrated that autophagy flux is inhibited after SCI in the mouse model, with obvious accumulation of autophagy markers LC3-II and autophagy substrate P62/SQSTM1 in ventral motor neurons (Liu et al., 2015b). Inhibition of autophagy flux is proved to be caused by rapid lysosomal dysfunction, contributing to accumulation of RIPK1, RIPK3, and MLKL after SCI, which were located in neurons destroyed by autophagy (Liu et al., 2018). The receptor-interacting protein kinases 1 (RIPK1) and the sequential activation of downstream RIPK3 and the mixed lineage pseudo-kinase MLKL regulates necroptosis which leads to neuronal and glial cell death after SCI (Liu et al., 2015a; Weinlich et al., 2017). As necroptosis markers, RIPK1, RIPK3, and MLKL are expressed in damaged spinal cord tissue and are negatively correlated with motor function recovery (Wang et al., 2018). Specific inhibition of RIPK1 kinase activity is found to block necroptosis and inflammation (Degterev et al., 2008). Thus, the interaction of lysosomal/autophagy and necroptosis may play a key role in improving neuronal survival and functional outcomes after SCI injury.

In this study, the effects of EA on improvement of lower limb function in rat modal would be investigated to explore its mechanism of autophagy flux and necroptosis regulated by lysosomal dysfunction after SCI, and to provide a potential effective therapy for SCI.

## Materials and Methods

### Animals Model

Female Sprague Dawley rats (220–250 g body weight), aged 10 weeks old, were obtained from the central animal of Heilongjiang Chinese Medicine university. Animals use and care protocols conform to guidelines by the International Council for Laboratory Animal Science (ICLAS) and Animal Care and Use Committee of China.

Rats were randomly divided into four groups: Sham group, SCI group, EA group, and EA + CQ group. The sham group received only laminectomy. Model group underwent T9-T11 SCI. Electro-acupuncture group (EA) performed Jia-Ji electro-acupuncture therapy 6 h after SCI. Electro-acupuncture + inhibitor chloroquine group (EA + CQ) received both EA and inhibitor CQ treatment after SCI. 6 h after the injury, the rats were treated with electro-acupuncture. At the same time, the rats were intraperitoneally injected with inhibitor chloroquine (CQ, C6628, United States, Sigma) at an injection dose of 50 mg/kg/d, until the rats were killed. The Sham group, Model group and EA group were injected daily with the same dose of normal saline as CQ. Rats in the SCI, EA and EA + CQ groups were further divided into four subgroups by various time points of treatment: 6 h, 1, 3, and 7 days. And 3 and 7 days subgroups were focus of subsequent studies.

Allen’s model was made by application of NYU Impactor M-III strike. After anesthesia with 10% chloral hydrate (3.0 mL/kg body weight) *via* intraperitoneal injection, a 5 cm longitudinal incision was made dorsally on the rats. Expose the T9-11 spinous processes, and resect the T10 lamina to create a gap around vertebral arch plate. Repeat extend the gap and remove the vertebral plate and T10 spinous process, and expose the intact spinal cord. For Sham group, only laminectomy was performed. The SCI model was then produced using a NYU Impactor M-III strike (a 10 g pouching rod of 2.5 mm diameter with drop of 50 mm and damage of 50 g⋅cm) at the T10 segment to create relatively serious injury (Verma et al., 2019). All SCI rats conformed to the following injury criteria: spinal cord ischemia and edema, formation of tail sway reflex, flicking of both body and legs, and the appearance of sluggish paralysis. After the strike was completed, marked the skin on both sides of the wound, and applied antibiotics (amoxicillin) to the incision site. Ensure adequate food and water, assist urination once in the morning and evening until the reflex of urination was restored, and massaged the abdomen of rats clockwise every day to prevent flatulence and difficulty in defecation.

Electro-acupuncture treatment method (EA): 6 h after injury, Jia-Ji electro-acupuncture therapy was performed. Operation method: The upper and lower spinous process gaps (proximal end at the top, distal end at the bottom) in the damaged area were selected to open points within 7 mm. After routine disinfection, inserted the needle into the spine with a needle tip to the lamina (depth is 13 mm). With Micro Plus type pulse electro-acupuncture (BioMedical Life Systems, Inc., Vista, CA, United States), the positive pole was connected to the upper needle handle, and the negative pole to the lower needle body on the same side. Selection parameters: Pulse wave type: continuous pulse waveform; Pulse repetition frequency: 100 Hz; Current output intensity: slight twitch of back muscle (about 1 mA). It lasted for 30 min, once a day.

### Behavioral Assessment

The Basso-Beattie-Bresnahan (BBB) scores was used to evaluate the functional recovery of the hind limbs in rats with SCI (Basso et al., 1995). The scale ranged from 0 to 21 (0 = complete hind limb paralysis, 21 = normal locomotion) for joint movement, gait and hind limb coordination, and fine motion of the toes. The higher the score, the better the motor function. The rats in each group were dynamically evaluated according to the 6 h, 1, 3, and 7 days after SCI. The rats were placed in the open field and moved freely for 5 min. Two trained observers scored performance of the rats blindly and recorded the average value (Hong et al., 2019).

### Hematoxylin-Eosin Staining and Nissl Staining

Rats in Sham, Model, and EA groups (*n* = 6) were subjected to deep anesthesia with 10% chloral hydrate (3.5 ml/kg, I.P.) at different time points. The rats were sacrificed by cutting off their heads quickly, and the spinal cord in the damaged area was quickly removed on the ice bag and then fixed with 4% paraformaldehyde. Paraffin-embedded lesion. Transverse paraffin sections (5 μm thick) were placed on slides. HE staining was performed for histopathological examination. The sections were incubated in 1% toluidine blue for Nissl staining and observed under light microscope.

### Immunohistochemistry

Rats in Sham, Model, EA, and EA + CQ groups were deeply anesthetized with 10% chloral hydrate and executed, then the injury section of spinal cord tissue were fixed in 4% paraformaldehyde. A 5μm thick of paraffin embedding spinal cord section was incubated with primary antibody 4°C overnight after dewaxing, transparency, hydrogen peroxide incubation, antigen repair, and blocking. The mixture was placed in room temperature for 30 min, rinsed with PBS for 3 times, incubated with secondary antibody at 37°C for 30 min. Finally, sections were incubated with DAB, hematoxylin redyeing, and mounted on coverslips. Photographic results were taken under a light microscope (400 ×) and quantitative assessment of immunohistochemical staining was performed by Image-Pro Plus 6.0 software. Primary antibodies: RIPK1 (1:100, RIPK1 Rabbit PolyClonal antibody, United States Proteintech), RIPK3 (1:40, Rabbit PolyClonal antibody, Beijing BIOSS), MLKL (1:200, Rabbit PolyClonal antibody, United States Affinity), LC3B (1:500, Rabbit polyclonal antibody, United States Proteintech), P62 (1:200, Rabbit polyclonal antibody, United States Proteintech). Second antibody: anti-rabbit (PV-6,001, Beijing Zhongshanjinqiao).

### Transmission Electron Microscopy

Rats in Sham, Model, and EA groups (*n* = 6) at 3 and 7 days time points were euthanized. The spinal cord tissues at the injured sites were removed, cut into small pieces of about 1 mm × 1.5 mm × 1.5 mm, and fixed with 2.5% glutaraldehyde for more than 24 h and then with 2% osmium acid for 2 h. After dehydration in acetone, the embedded spinal cord sections were stain with toluene ammonia blue to select required sites for observation. Sweden made LKB-III ultra-thin slicing machine was used to section with the thickness of about 500A. The samples were stained with uranium acetate and lead nitrate and observed by transmission electron microscope.

### Real-Time Quantitative Polymerase Chain Reaction (qRT-PCR)

The spinal cord tissue at the damaged site in each group was quickly removed and put into liquid nitrogen, and then transferred to the −80°C refrigerator for use. Total RNA was isolated using BufferRL1. The mRNA expression of RIPK1, RIPK3, MLKL were measured using the RT-qPCR system. The reverse transcription system was as follows: RNase Free dH2O: 10 μl, 5X PrimeScript Buffer 2 (for Real Time): 4 μl, RNA: 4 μl, RT Primer Mix (4 ×): 1 μl, Primescript RT Enzyme Mix I: 1 μl. 42°C for 15 min and 85°C for 5 s for reverse transcription. PCR reaction solution was as follows: TBGreen Premix Ex Taql (Tli RNaseH Plus) (2 ×): 10 μl, PCR Forward Primer(10 μM): 0.8 μl, PCR Reverse Primer (10 μM): 0.8 μl, RT reaction solution (cDNA): 2 μl, sterilized water: 6.4 μl, Total: 20 μl. Target gene primer sequence: RIPK1 (For 5′-GAGGAGGAAAGGAAGCGAAG-3′, Rev 5′-TGACTGGTTGTGCTGGGATA-3′) 113 bp, RIPK3 (For 5′-GAGTGGGACTACGTGTACGG-3′, Rev 5′-CAGCAGAACA TTGGAGGGCT-3′) 204 bp, MLKL(For 5′-AGCCTCCCCAGT GACATTAC-3′, Rev 5′-GCCCACAGTAGCAAACTTCC-3′) 120 bp, β-actin (For 5′-CCTGTGGCATCCATGAAACTAC-3′, Rev 5′-CCAGGGCAGTAATCTCCTTCTG-3′) 150 bp. PCR amplification procedure followed by 40 cycles of 95°C for 5 s, 60°C for 30 s. Delta-delta Ct (ΔΔCt) method was used to calculate the relative expression of the target gene. PrimeScript RT reagent Kit with gDNA Eraser (Perfect Real Time) (Japan, Takara), TB Green Premix Ex Taq II (Tli RNaseH Plus) (Japan, Takara).

### Western Blot Assay

Rats in Sham, Model, EA, and EA + CQ groups (*n* = 6) at 3 and 7 days time points were euthanized, and the spinal cord tissues at the damaged sites were removed rapidly and homogenized in lysis buffer. With PMSF15 ml, 12,000 rpm, centrifuged for 5 min, the supernatant was absorbed, and the protein concentration was determined by BCA. Loading sample for electrophoresis and then transferred the protein to the PVDF membrane. After blocking for 1 h, the PVDF membrane was incubated with the primary antibody at 4°C overnight, as shown below: LC3B (1:2,000, Rabbit polyclonal antibody, United States Proteintech), P62 (1:1,000, Rabbit polyclonal antibody, United States Proteintech), RIPK1 (1:1,000, RIPK1 Rabbit PolyClonal antibody, United States Proteintech), RIPK3 (1:2,000, Rabbit polyclonal antibody, Beijing BIOSS), MLKL (1:1,000, Rabbit polyclonal antibody, United States Affinity), β-actin (1:5,000, mouse monoclonal antibody, United States Proteintech). The membrane were washed 3 times with Tris-buffered saline Tween, and incubated with secondary antibody at 37°C for 1 h as shown below: HRP-Goat-Anti-Rabbit IgG(H + L) (1:5,000, United States Proteintech), HRP-Goat-Anti-Mouse IgG(H + L) (1:5,000, United States Proteintech). The immunoreactive bands were visualized by ECL chemiluminescence. The grayscale values of bands were quantified by Image Lab 5.2.1 software, and the relative expression of protein was calculated according to the ratio of target protein to β-actin.

### Statistical Analyses

SPSS 25.0 software was used for statistical analysis. The data were presented as mean ± standard deviation (± SD). One-way analysis of variance (ANOVA) was used for comparison between groups. For further comparison between groups, the least significant difference (LSD) test was used for those with homogeneous variance, and Dunnett’s T3 test was used for those with heterogeneous variance. *P* < 0.05 was considered statistically significant.

## Results

### Electro-Acupuncture Improved Motor Function

**FIGURE 1:**
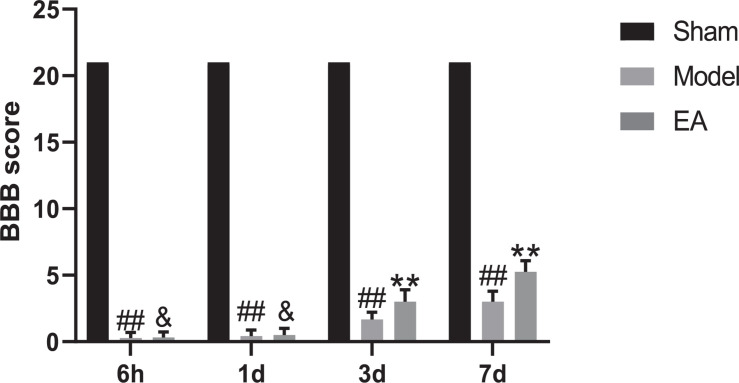
BBB scores of four different time points. Data are expressed as the mean ± SD (*n* = 6 rats/group). ^##^*P* < 0.01, vs. Sham group; ^&^*P* > 0.05, **P < 0.01, vs. Model group. BBB: Basso, Beattie and Bresnahan rating scale, used to evaluated hind limb locomotor. Sham, laminectomy only; Model, spinal cord injury; EA, electro-acupuncture.

BBB scale scores were used to assess movement of hind limb. The first part mainly evaluates the range of motion of joints, with a score of 0–7. The second part mainly evaluates the gait and hind limb coordination ability, with a score of 8–13. The third part mainly assessed the fine movement of the toes, with a score of 14–21 points. After SCI, the rats were paralyzed in the hind limbs, without any movement phenomenon, with reduced diet and water, accompanied by urinary retention, and entered the stage of spinal cord shock, with a score of 0 point. The evaluation results of Sham group were all scored 21 points at the four time points of 6 h, 1, 3, and 7 days, and the rats walked as usual, indicating no dyskinesia. The scores of Model group at each time point after SCI were significantly lower than Sham group (*P* < 0.01), which means a significant movement disorder following SCI. After EA treatment, there was little change in the score at 6 h and 1 day, and no statistical significance was found compared with the Model group (*P* > 0.05). The scores were significantly greater in the EA group compared with the SCI group on days 3 and 7 after injury (*P* < 0.01; Figure 1). Motor dysfunction of the hind limbs could be improved in the rats with EA treatment at 3 and 7 days.

### Effects of Electro-Acupuncture on Histological Changes in Spinal Cord

**FIGURE 2:**
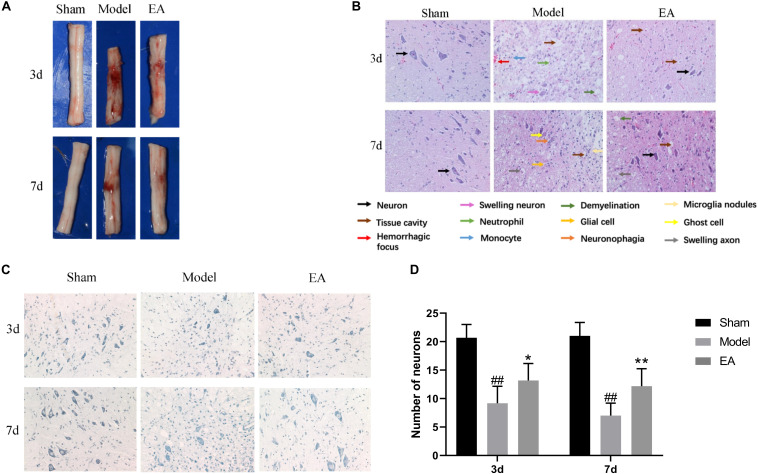
Effect of EA on histological changes in spinal cord at 3 and 7 days. **(A)** Spinal cord tissue appearance. EA could promote bleeding absorption, reduce edema and protect spinal cord. **(B)** HE stained images of spinal cord sections (200×). EA could improve the pathological changes. Arrows with different colors means different structure and pathological changes, listed with the image. In model group, neuron disappear compared to Sham. Tissue cavity, hemorrhagic focus, swelling neuron, neutrophil, monocyte and demyelination could be seen in Model group at 3 day. Glial cell, ghost cell, neuronophagia, swelling axon and microglia nodules are shown in Model group at 7 day. While after EA treatment, several neurons could be seen, the damage tissue and inflammation factors reduce. **(C)** Nissl stained images of motor neuron (200×). The number of motor neuron could increase after EA treatment. **(D)** Number of anterior horn motor neurons in each group. Data are expressed as the mean ± SD (*n* = 6 rats/group). ^##^*P* < 0.01, vs. Sham group; *P < 0.05, **P < 0.01 vs. Model group.

At the time points of 3 and 7 days, the appearance and structure of the spinal cord tissues in Sham group was normal. The hematoma after SCI in Model group was most severe on 3 day. After the EA treatment, the hematoma area became significantly smaller at each time point (Figure 2A).

In the SCI group on 3 day, pathological change was most obvious with gray structure disorder, necrosis and inflammation worsen, a large number of neurons lost, the serious neurons swelling, neutrophils, and monocytes infiltration, white matter demyelinating and scattered focus of hemorrhage. Compared with the Model group, the EA group had fewer necrotic cavities, improved neuronal swelling, more orderly gray matter structure, less neuron loss, and improved inflammatory cell infiltration. At the time point 7 days after SCI, the loss of gray matter neurons in the Model group was still significant, the necrotic cavity gradually expanded, which showed the ghost cell of dead neurons, the decrease of inflammatory cells compared with that at 3 day, and a large number of glial cells proliferated. In this figure, the image of neuronophagia and nodular microglia could be seen. There are numerous voids in the white matter, marked demyelinating changes, and the ends of axons swell into a spherical shape. The hemorrhagic lesion was reduced compared with that of 3 day, while the above pathological symptoms were improved after treatment in the EA group. The gray matter structure was relatively complete, the nerve fibers were arranged in a more orderly manner, the boundary between gray matter and white matter could be observed, the number of neurons was more, the number of cavities was less, and the inflammatory cell infiltration was improved (Figure 2B).

Nissl staining was used to observe the morphology and number of motor neurons in the anterior horn of spinal cord in each group. In Sham group, the anterior horn motor neurons were orderly arranged without significant reduction, deep blue staining. At 3 and 7 days time points, there were statistically significant reducing in the number of anterior angular motor neurons in Model group compared to Sham group (*P* < 0.01). Although the number of neurons in the EA group decreased, the number of neurons in the EA group was still higher than that in the Model group, and the staining was relatively deeper. The comparison between the two groups was statistically significant (3 day: *P* < 0.05, 7 day: *P* < 0.01) (Figures 2C,D).

### Electro-Acupuncture Inhibited the Expression of Necroptosis Markers RIPK1, RIPK3, and MLKL

**FIGURE 3:**
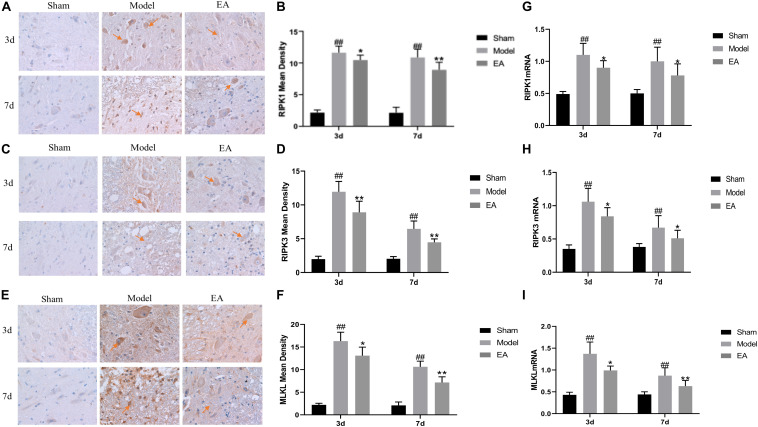
Effects of EA on the expression of necroptosis markers. **(A–F)** Immunohistochemistry results of RIPK1, RIPK3, and MLKL in spinal cord of rats in each group. RIPK1, RIPK3, and MLKL were brown-yellow positive, mainly expressed in neuron and glial cells (red arrow). **(B,D,F)** Average OD of RIPK1, RIPK3, and MLKL in each group. After EA treatment, these necroptosis markers reduced compared to Model. Data are expressed as the mean ± SD (*n* = 6 rats/group). **(B)**:^##^*P* < 0.01, vs. Sham group; *P < 0.05, **P < 0.01, vs. Model group; **(D)**:^##^*P* < 0.01, vs. Sham group; **P < 0.01, vs. Model group. **(F)**: ^##^*P* < 0.01, vs. Sham group; *P < 0.05, **P < 0.01, vs. Model group. **(G–I)** Quantification of RIPK1, RIPK3 and MLKL mRNA by qRT-PCR. **(G)**: ^##^*P* < 0.01, vs. Sham group; *P < 0.05, vs. Model group. **(H)**: ^##^*P* < 0.01, vs. Sham group; *P < 0.05, vs. Model group. **(I)**: ^##^*P* < 0.01, vs. Sham group; *P ≤ 0.05, **P < 0.01, vs. Model group.

RIPK1, RIPK3, and MLKL were mainly expressed in the cytoplasm of neurons and glial cells. The average OD value of RIPK1, RIPK3, and MLKL in the Model group was significantly higher than that of the Sham group at 3 and 7 days after injury (*P* < 0.01). After EA treatment, the mean OD value of RIPK1, RIPK3, and MLKL decreased, and the comparison between the EA group and the Model group was statistically significant at the time points of 3 and 7 days (RIPK1, 3 day: *P* < 0.01, 7 day: *P* < 0.05; RIPK3, 3 day/7 day: *P* < 0.01; MLKL, 3 day: *P* < 0.05, 7 day: *P* < 0.01. Figures 3A–F). It may illustrate that the expression level of RIPKI, RIPK3, and MLKL increased after SCI, and the EA had the effect of inhibiting the necroptosis factors.

qRT-PCR results showed that the expression of RIPK1, RIPK3, and MLKL mRNA in the Model group was significantly higher than that in the Sham group at 3 and 7 days after SCI (*P* < 0.01). After treatment, the expression level of those mRNA in the EA group was decreased, and the difference between the EA group and the Model group was statistically significant at 3 and 7 days time points (RIPK1, RIPK3, *P* < 0.05; MLKL, 3 day: *P* ≤ 0.05, 7 day: *P* < 0.01. Figures 3G–I). This result is consistent with immunohistochemistry results.

### Electro-Acupuncture Enhanced Autophagy Flux and Inhibited Necroptosis

#### Electro-Acupuncture Increased Numbers of Autolysosomes

**FIGURE 4:**
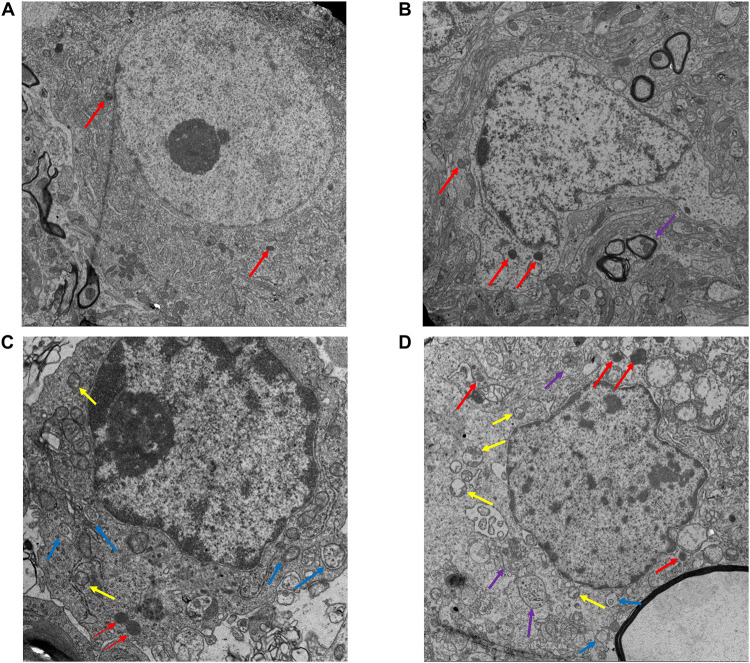
Electron microscopy results of the spinal cord tissue sections after EA. **(A)** Ultrastructure of the sham group (13,000×). Nuclei is intact (red arrow), with obvious nucleoli. The myelin sheath is arranged around the axon in concentric circles. No autophagosome is found. **(B)** Ultrastructure of spinal cord tissue after injury in 3 day (18,000×). In model group, the myelin sheath and the nucleus shrinks and several lysosomes (Red arrow) and autolysosomes (purple arrow) are shown. **(C)** EA + CQ group in 3 day (44,000×). Autophagosomes accumulated significantly. Several active lysosomes could be seen. **(D)** EA group in 3 day (18,000×). The number of lysosomes, autophagosomes and autolysosomes increased in EA group, especially active lysosomes. Yellow arrow: secondary lysosome (active lysosome). Blue arrow: autophagosomes with two membranes. Red: primary lysosomes. Purple: autolysosomes with single membrane.

Electron microscopy was used to observe the structure changes of spinal cord tissue after injury. In the Sham group, the nuclei were intact and uniformly stained, the number and structure of organelles in the cytoplasm were normal, the myelin sheath was arranged in concentric circles around the axons, and microfilaments and microtubules could be seen in the axons in regular arrangement. No axonal denaturation and granular disintegration were observed, no autolysosome was found, and several primary lysosomes could be seen (Figure 4A). On 3 day after SCI, the cytoplasm of neurons in the spinal cord tissue had sustained extensive edema, the mitochondria were swollen significantly, the myelin lamina was broken, the rough endoplasmic reticulum was loose, the number of lysosomes increased (Figure 4B). In the EA + CQ group, autophagosomes increased much more than that in EA group, while only several autolysosomes could be seen (Figure 4C). CQ destroyed lysosome function by inhibiting the fusion of lysosome and autophagosomes, leading to the accumulation of autophagosomes, and blockage of autophagy flux. The number of lysosomes, especially secondary lysosomes/active lysosomes, autophagosomes, and autolysosomes in the EA group was greater than that in the model group (Figure 4D). This results may illustrate that EA have effects on increasing the numbers and function of lysosomes and autolysosomes and enhancing autophagy flux.

#### Lysosomes Inhibitor CQ Eliminated Effect of EA on Motor Function Improvement and Histological Changes

**FIGURE 5:**
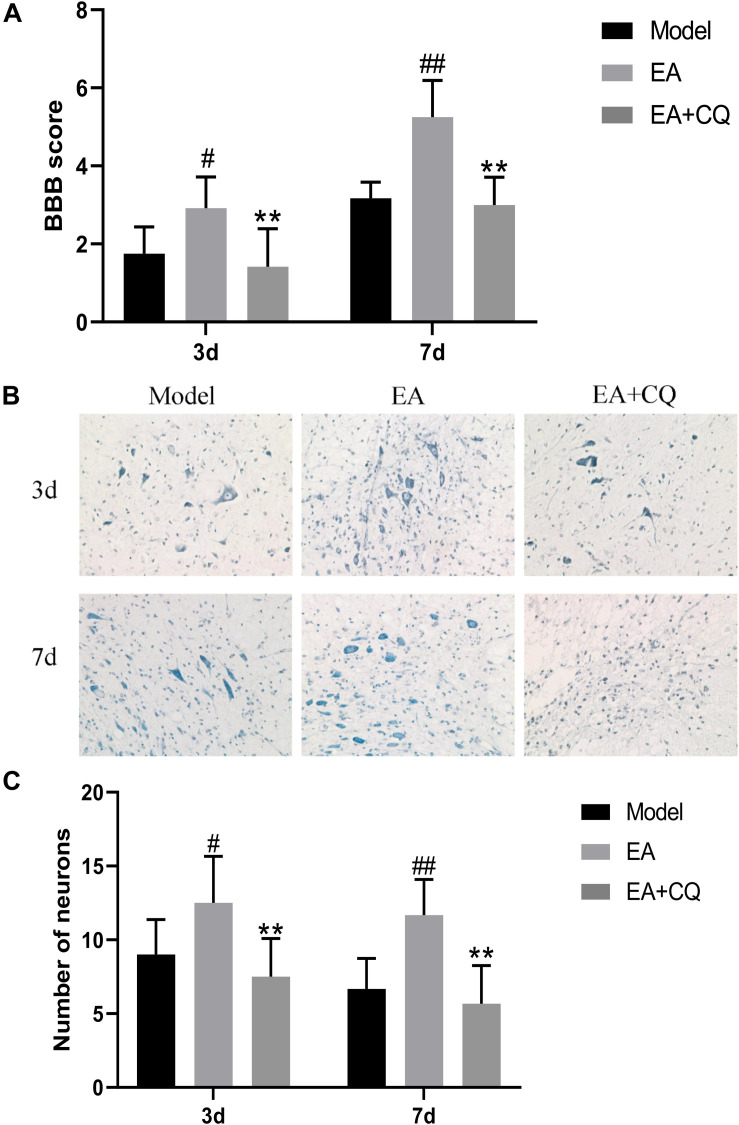
**(A)** BBB scores of neurological function recovery after injection of CQ. Data are expressed as the mean ± SD (*n* = 6 rats/group). ^#^*P* < 0.05, ^##^*P* < 0.01, vs. Model group. **P < 0.01, vs. EA group. Model, spinal cord injury; EA, electro-acupuncture; EA + CQ, electro-acupuncture with injection of inhibitor CQ. **(B)** Nissl staining results of spinal cord tissue after inhibitor CQ injection (200 ×). **(C)** Number of anterior horn motor neuron. Data are expressed as the mean ± SD (*n* = 6 rats/group). ^#^*P* < 0.05, ^##^*P* < 0.01, vs. Model group. **P < 0.01, vs. EA group.

The EA group scored higher than the Model group on both 3 and 7 days, with statistically significant differences (*P* < 0.05, *P* < 0.01, Figure 5A). However, after injection of lysosomes inhibitor CQ, the EA + CQ group had lower scores on 3 and 7 days than the EA group significantly (*P* < 0.01), indicating that CQ could partially eliminate the therapeutic effect of EA on improvement of motor function.

At the time points of 3 and 7 days after surgery, the number of neurons in the Model group was significantly reduced, while neurons relatively increased after treatment in the EA group, statistically significant compared with the Model group at each time point (*P* < 0.05, *P* < 0.01). However, the neurons in the EA + CQ group decreased after injection of inhibitor CQ at 3 and 7 days compared with that in the EA group (*P* < 0.01), indicating that injection of inhibitor CQ could partially eliminate the therapeutic effect of EA and reduce the number of neurons (Figures 5B,C).

#### Electro-Acupuncture Promoted Autophagy Flux Blocked by Lysosomes Inhibitor

**FIGURE 6:**
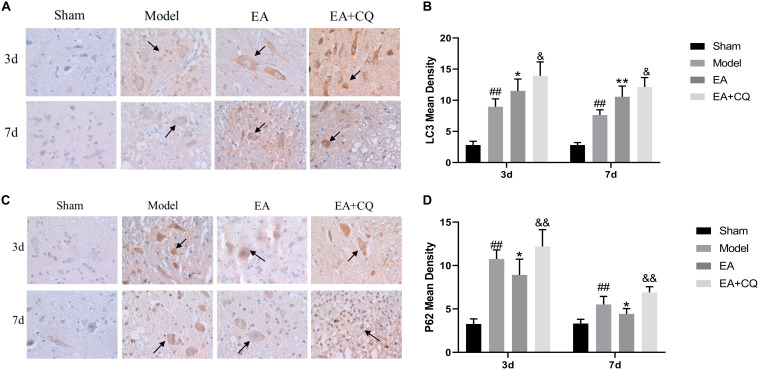
Immunohistochemistry results on the expression of LC3 and P62. **(A)** Immunohistochemical staining results of LC3 in rats in each group (400×). LC3 is brown staining (black arrow). **(B)** Mean optical density of LC3 in each group (IOD/Area). ^##^*P* < 0.01, vs. Sham; *P < 0.05, **P < 0.01, vs. Model; ^&^*P* < 0.05, vs. EA. **(C)** Immunohistochemical staining results of P62 in each group (400 ×). P62 is brown staining (black arrow). **(D)** Mean optical density of P62 in each group (IOD/Area). ^##^*P* < 0.01, vs. Sham; *P < 0.05, vs. Model; ^&&^*P* < 0.01, vs. EA. Data are expressed as the mean ± SD (*n* = 6 rats/group).

Immunohistochemistry results showed that LC3 and P62 were mainly expressed in cytoplasm of neurons and glial cells. At postoperative 3 and 7 days, the mean density value of LC3 and P62 in Model group were significantly higher than Sham group (*P* < 0.01). After treatment at 3 and 7 days, LC3 in the EA group increased significantly compared with the Model group (*P* < 0.05, *P* < 0.01), while the value of P62 in EA group was lower than that in Model group (*P* < 0.05). The mean density value of LC3 and P62 both increased in EA + CQ group at each time point after injection of inhibitor CQ, compared to that in EA group (*P* < 0.05, *P* < 0.01). The results showed that the expression of LC3 increased after SCI, which may be related to the decreased autophagosomal degradation caused by lysosomal dysfunction. While electro-acupuncture could promote the expression of LC3 and increase the formation of autophagosome. After SCI, the expression level of autophagy degradation substrate P62 was increased, and the acupuncture could reduce the expression of P62, promote autophagy degradation, and accelerate autophagy flux. On the basis of the acupuncture treatment, lysosomal inhibitor CQ was injected to enhance the expression of LC3 and P62, leading to excessive accumulation of autophagosomes and blocking of autophagy flux (Figure 6).

**FIGURE 7:**
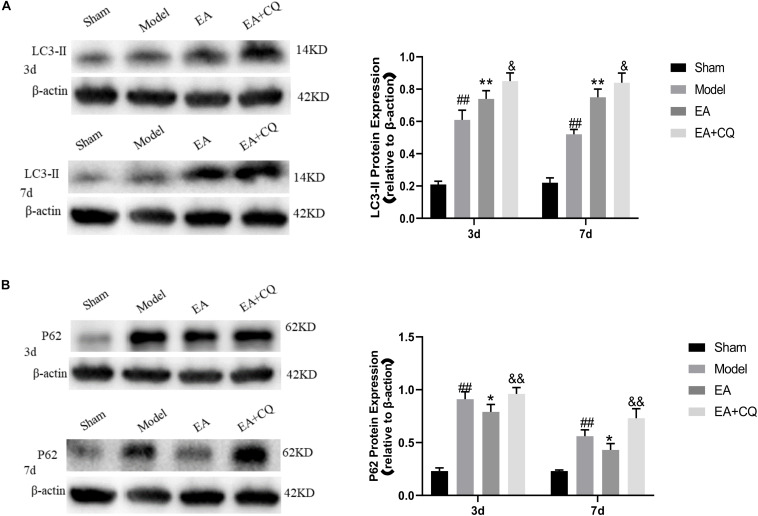
Western blot results of the expression of LC3-II and P62 protein. **(A)** Expression of LC3-II in each group (LC3-II/β-actin). ^##^*P* < 0.01, vs. Sham; **P < 0.01, vs. Model; ^&^*P* < 0.05, vs. EA. **(B)** Expression of P62 protein in each group (P62/β-actin). ^##^*P* < 0.01, vs. Sham; *P < 0.05, vs. Model; ^&&^*P* < 0.01, vs. EA.

It is further verified by Western Blot. LC3-II protein expression in Model group was significantly higher than that in Sham group at 3 and 7 days postoperatively (*P* < 0.01). After EA treatment, the expression level of LC3-II protein increased, which was statistically significant compared with the Model group at the time points of 3 and 7 days (*P* < 0.01). However, LC3-II protein in the EA + CQ group continued to increase after injection of inhibitor CQ, and the difference between the EA group and the EA group was statistically significant (*P* < 0.05). These results indicate that the expression of LC3-II protein increases and autophagy increases after SCI, which may be caused by the blockage of autophagy flux. The continued increase of LC3-II protein expression after acupuncture treatment may be related to the promotion of autophagy. The inhibitor CQ was injected on the basis of the electro-acupuncture treatment, resulting in the further increase of LC3-II protein expression and a large amount of autophagosome accumulation (Figure 7A).

P62 protein expression in the Model group was significantly increased compared with that in the Sham group (*P* < 0.01), while it was decreased in EA group, which was statistically significant compared to Model group at 3 and 7 days after treatment (*P* < 0.05). After injection of inhibitor CQ, the expression of P62 protein in the EA + CQ group was higher than that in the EA group at each time point (*P* < 0.01). These results indicated that P62 protein expression increased after SCI. After EA treatment, the expression of P62 protein was decreased, autophagy degradation was promoted, and autophagy flux was enhanced. The inhibitor CQ could partly eliminated the effect of the EA on reducing P62 protein expression, but promoted its increase (Figure 7B).

#### Lysosomes Inhibition Eliminated the Effect of Electro-Acupuncture on Reducing Necroptosis Markers Expression

**FIGURE 8:**
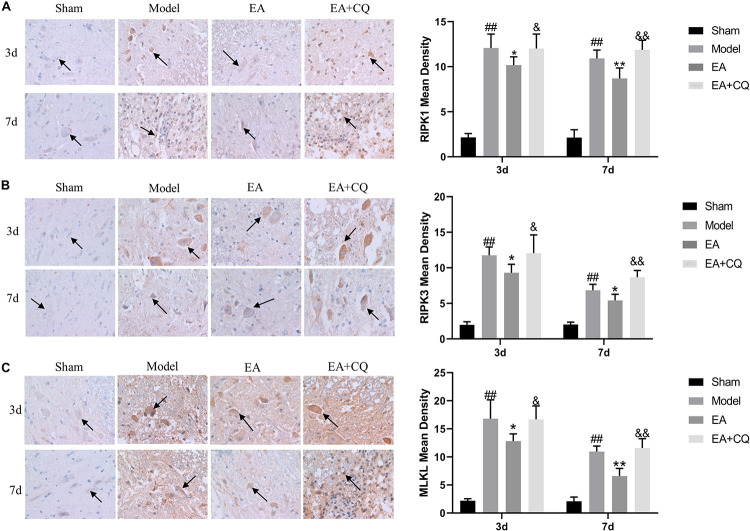
Immunohistochemistry results on the expression of RIPK1, RIPK3, and MLKL. RIPK1, RIPK3 and MLKL were brown-yellow positive, mainly expressed in neuron and glial cells (black arrow). **(A)** Immunohistochemistry results of RIPK1 in rats in each group (400×). Mean optical density of RIPK1 in each group (IOD/Area). ^##^*P* < 0.01, vs. Sham; *P < 0.05, **P < 0.01, vs. Model; ^&^*P* < 0.05, ^&&^*P* < 0.01, vs. EA. **(B)** Immunohistochemistry results of RIPK3 in rats in each group (400×). Mean optical density of RIPK3 in each group (IOD/Area).^##^*P* < 0.01, vs. Sham; *P < 0.05, vs. Model; ^&^*P* < 0.05, ^&&^*P* < 0.01, vs. EA. **(C)** Immunohistochemistry results of MLKL in rats in each group (400×). Mean optical density of MLKL in each group (IOD/Area). ^##^*P* < 0.01, vs. Sham; *P < 0.05, **P < 0.01, vs. Model; ^&^*P* < 0.05, ^&&^*P* < 0.01, vs. EA.

At 3 and 7 days after surgery, the average optical density value of RIPK1, RIPK3, MLKL in the EA group were lower than that of the Model group (RIPK1, MLKL: *P* < 0.05, *P* < 0.01; RIPK3: *P* < 0.05), while the value of them in EA + CQ group increased after the injection of inhibitor CQ, and the comparison between the EA + CQ group and the EA group was statistically significant at 3 and 7 days after surgery (*P* < 0.05, *P* < 0.01, Figure 8). These results indicated that EA treatment could inhibit the formation of necroptosis factor RIPK1, RIPK3, MLKL, reduce the formation of necrotic complex, and protect the remaining neurons. However, after injection of inhibitor CQ, the effect of EA on necroptosis factors reduction was partially eliminated, so as to increase the value of necroptosis factor and reduce the survival rate of neurons.

**FIGURE 9:**
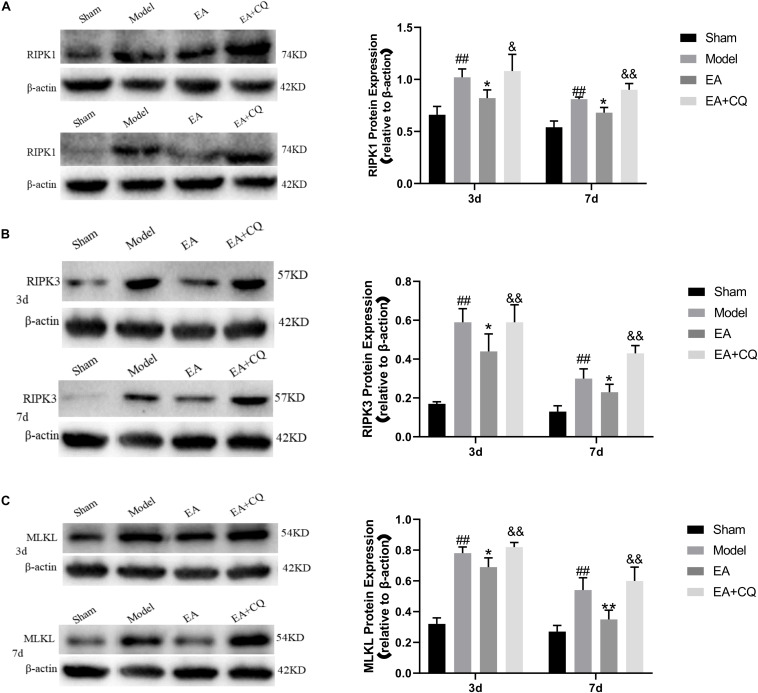
Western blot results on the expression of RIPK1, RIPK3, and MLKL. **(A)** Expression of RIPK1 protein in each group (RIPK1/β-actin). ^##^*P* < 0.01, vs. Sham; *P ≤ 0.05, vs. Model; ^&&^*P* < 0.01, ^&^*P* < 0.05, vs. EA. **(B)** Expression of RIPK3 protein in each group (RIPK3/β-actin). ^##^*P* < 0.01, vs. Sham; *P < 0.05, vs. Model; ^&^*P* < 0.05, ^&&^*P*< 0.01, vs. EA. **(C)** Expression of MLKL protein in each group (MLKL/β-actin). ^##^*P* < 0.01, vs. Sham; *P < 0.05, **P < 0.01, vs. Model; ^&&^*P* < 0.01, vs. EA.

The expression of RIPK1, RIPK3, and MLKL in the Model group was significantly increased compared with that in the Sham group at 3 and 7 days (*P* < 0.01), while it was decreased after treatment in the EA group. At the time points of 3 and 7 days, the difference was statistically significant compared with that in the Model group (RIPK1: *P* ≤ 0.05; RIPK3: *P* < 0.05; MLKL: *P* < 0.05, *P* < 0.01). Compared with the EA group, RIPK1, RIPK3, and MLKL protein expression in the EA + CQ group increased significantly at each time points after injection of inhibitor CQ (RIPK1: *P* < 0.01, *P* < 0.05; RIPK3: *P* < 0.05, *P* < 0.01; MLKL: *P* < 0.01, Figure 9). The results show that RIPK1, RIPK3, and MLKL protein expression increased after SCI, and EA treatment could reduce these necroptosis factors expression and necrosis complex formation. Inhibitor CQ could partly eliminate the effect of EA on reducing RIPK1, RIPK3, and MLKL protein expression, instead promoting its elevation, which is available in the formation of necrotic complexes and exacerbates neuronal necrosis.

## Discussion

Clinically, EA as a traditional Chinese medicine therapy has been widely used to improve the prognosis of spinal cord and neuromuscular injury. Electro-acupuncture at Jia-Ji points is a combination of acupuncture effect and electric field effect on injured spinal cord after SCI. In this study, we investigated the molecular biological mechanism of EA treatment on improvement of lower hind motor and autophagy regulation of necroptosis. Our data showed that lysosome function was inhibited after SCI with increasing the expression of LC3-II and P62. LC3-II reflects the number of autophagosomes and P62 is autophagy cargo protein. The number of autophagosomes and the degradation of autophagy cargo protein increased after EA treatment, which proved that the electro-acupuncture has the effect of enhancing autophagy flux. Consistent with previous study (Liu et al., 2018), we found that necroptosis markers were accumulated after SCI in neurons with inhibiting of autophagy flux and lysosomal damage. The accumulation of RIPK1, RIPK3, and MLKL were inhibited in neurons and glial cells by using Jia-Ji EA treatment, which may reduce necroptosis. Besides, the lysosome inhibitor CQ partially eliminated the therapeutic effect of EA on promoting autophagy flux and inhibiting necroptosis. This study suggests that Jia-Ji EA treatment may promote the recovery of motor function through the mechanism of accelerating autophagy flux and alleviating necroptosis.

Rodent models have become standard in the past 30 years and have been widely used in SCI research (Kwon et al., 2015). Rats are the best choice and the most widely used due to the limit field of vision of mice (Verma et al., 2019). In rat model of SCI, we observed that there was a significant improvement on motor function after EA treatment compared to Model group at 3 and 7 days time points (*P* < 0.01). The improvement of hind limb motor function was not obvious after treatment for 6 h or 1 day. So 3 and 7 days time points were selected as time points in the following study. It is further proved by the histological changes. At the time points of 3 and 7 days, the appearance and structure of the spinal cord tissues in the Sham group were normal, while the hematoma in the spinal cord attack area in the Model group was obvious. However, the hematoma was the most severe in 3 day, indicating that the vascular hemorrhage of the spinal cord after external impact caused congestive necrosis, leading to further aggravation of tissue ischemia. After EA treatment, the range of hematoma in EA group at each time point became smaller and the color became lighter. Spinal cord structure was disorder at 3 day after SCI in Model group, with nerve fibers arranged loosely, flake bleeding lesions, the massive death of neurons, retained a large number of neurons die cavity. These pathology changes are associated with cells and necrotic neurons apoptosis, known as vacuolization of neurons. While at 7 day after SCI, inflammatory cells infiltration reducing, hyperplasia of glial cells, and phagocytosis phenomenon of neurons could be seen on HE stain, suggests that microglia have the effect of the removal of necrotic neurons, leaving ghost necrotic neurons observed. These pathological findings are consistent with previous reports (Alexander and Popovich, 2009; Mortazavi et al., 2015; Quadri et al., 2020). The Nissl body is a marker of the functional state of neurons. From Nissl staining, we found that the number of residual neurons in the Model group increased after EA treatment (*P* < 0.05), indicating that the death of neurons was reduced. The results showed that the EA could improve the microcirculation and inhibit neuronal death, which may be related to the improvement of motor function at 3 and 7 days time points.

Necroptosis is a regulatory necrosis activated downstream of tumor necrosis factor receptor 1 (TNFR1), dependent on the activity of receptor interaction protein kinases 1 (RIPK1) and 3 (RIPK3), and recruits mixed lineage pseudo-kinase MLKL to form necrosome complex (Necrosome), mediating cell rupture and death (Vandenabeele et al., 2010). The accumulation of necroptosis markers RIPK1, RIPK3, and MLKL was found after SCI, which may contribute to neuronal and glial cell death (Liu et al., 2015a). The RIPK1 inhibitor NEC-1 and the MLKL inhibitor NSA was found to inhibit the expression of necrosis factor and improve the lower limb motor function (Wang et al., 2014, 2018). In our results, RIPK1, RIPK3, and MLKL were mainly expressed in the cytoplasm of neurons and glial cells, but also shown in the intercellular substance, possibly because necroptosis factors in the cytoplasm were released into the intercellular after cell rupture. The mean optical density values of spinal cord tissues RIPK1, RIPK3, and MLKL in the Model group were significantly higher than those in the Sham group at 3 and 7 days after surgery (*P* < 0.01). This indicated that necrotic complexes accumulated in large quantities after SCI, while the average optical density values of RIPK1, RIPK3, and MLKL were decreased at all-time points after treatment in the EA group (*P* < 0.05). It may illustrate that EA could inhibit necrosome accumulation and reduce necroptosis of neuron.

As an important and conserved lysosomal degradation pathway, autophagy is thought to be involved in many physiological and pathological processes. It has been shown that inhibiting autophagy may have a neuroprotective effect (Siracusa et al., 2016; Wei et al., 2016). Autophagy flux is a dynamic process involving the formation, transmission, and degradation of autophagosomes. The increase of autophagosomes alone does not mean the enhancement of autophagy, and the accumulation of autophagosomes may occur due to the reduction of autophagosome degradation or the inability to degrade with the increase of autophagosome formation, thus inhibiting autophagy flux. The beneficial or harmful functions of autophagy may depend on the induction or inhibition of autophagy flux after central nervous system injury. Smooth autophagy flux often leads to cell protection, while blocked autophagy flux contributes to cell death. In our results, LC3 and P62 were mainly expressed in the cytoplasm of neurons and glial cells, consistent with the location of necrotic apoptotic factors. Average optical density values of LC3 and P62 were significantly higher than that of Sham group at 3 and 7 days postoperatively (*P* < 0.01). Besides, the expression peak of LC3 and P62 at 3 day was in accordance with that of necroptosis factors. The increase of LC3 and P62 after SCI indicates the blockage of autophagy flux, which may be caused by dysfunction of lysosomes on fusion and degradation of autophagosome after injury. The average optical density value of LC3 in the EA group was higher than that of the Model group at all-time points (*P* < 0.05), while the average optical density value of P62 was lower than that of the Model group (*P* < 0.05), indicating that EA treatment promoted the increase of LC3, reduced the expression of P62, and accelerated autophagy flux. LC3-II is generally considered as an autophagosome marker, because the content of LC3-II reflects the number of autophagosomes. However, LC3-II may not be able to estimate autophagy activity. Not only autophagy activation, but also inhibition of autophagosomal degradation greatly increases the content of LC3-II. The degradation of autophagy substrate P62/SQSTM1 is another marker widely used to evaluate autophagy activity, which can recruit ubiquitin substrates into autophagosome and degrade with it (Yoshii and Mizushima, 2017). When autophagy flux is high, the level of p62 protein decreases. Conversely, a reduction in autophagy flux may lead to P62 protein accumulation. Moreover, it was observed by electron microscopy that the number of lysosomes, autophagosomes and autolysosomes were increased compared with the model group after SCI was treated by EA. This result also indicates that EA could increase autophagy flux and promote autophagy degradation, consistent with the results that increase of LC3 expression and decrease of P62 expression mentioned before. More active lysosomes appeared after EA treatment, indicating Jia-Ji EA may stimulate lysosome function. The mechanism of EA on increasing autophagy flux may be related to the increase of autophagosome formation and the improvement of lysosomal function. However, the mechanism of Jia-Ji EA on increasing the number of lysosomes and enhancing its function still needs further study.

Lysosome function plays an important role in promoting autophagy flux. When autophagy flux is blocked due to lysosome dysfunction, the accumulation of autophagosomes would possibly lead to cell death (Lipinski et al., 2015). The autophagosome can be used as a basis to assemble necrotic complexes (Goodall et al., 2016). RIPK1 is recruited by P62 [the ZZ domain of P62 can interact with RIPK1 (Puissant et al., 2012)] to the autophagosome, which activates the assembly of necrosome and further mediates cell death. After inhibition of lysosomal function, autophagosomes accumulated, and could not be successfully degraded, contributing to the increase of LC3 and P62 contents. It has been previously demonstrated that autophagy flux was inhibited after SCI and treatment with rapamycin could attenuated accumulation of P62 by improving lysosomal function and autophagy flux (Liu et al., 2018). To further explore the effect of autophagy flux on rat hind limb function, lysosome inhibitor chloroquine CQ was applied. Many studies supported lysosomal luminal alkalinizer chloroquine (CQ) as an inhibitor of autophagy flux in rats with SCI (Hu et al., 2017; Zhang et al., 2017). CQ could improve the *pH*-value of lysosomes and then destroy the function of lysosomes, inhibit fusion of lysosomes and autophagosomes, thus leading to the accumulation of autophagosomes and blockage of the autophagy flux. BBB score and Nissl staining were observed after injecting CQ on the basis of EA treatment. At the time points of 3 and 7 days, BBB score was lower than that of the EA group (*P* < 0.01). The staining of motor neurons in the anterior horn was relatively light and the number of them was decreased compared with that of the EA group (*P* < 0.01), indicating that injection inhibitor CQ partially eliminated the therapeutic effect of EA, reduced the number of motor neurons in the anterior horn, and aggravated dysfunction of hind limbs in rats.

After treatment, the average optical density values of RIPK1, RIPK3, and MLKL in the EA group were lower than those in the Model group at each time point (*P* < 0.05), indicating the inhibitory effect of EA on necroptosis. After the injection of inhibitor CQ, the average optical density values of LC3, P62, RIPK1, RIPK3, and MLKL in the EA + CQ group increased at each time point, which was statistically significant compared with the EA group at each time point (*P* < 0.05). These results indicated that CQ partially eliminated the effect of EA on promoting autophagy flux and inhibiting necrotizing apoptosis. The results above were further confirmed by Western blot. Protein expression levels of LC3, P62, RIPK1, RIPK3, and MLKL were significantly higher than that of Sham group at 3 and 7 days after surgery (*P* < 0.01), indicating autophagy flux blocked and necrotic protein expression enhanced after SCI. The expression of LC3-II was all higher than that in the Model group at each time point (*P* < 0.01) after EA treatment, while the protein expression levels of P62, RIPK1, RIPK3, and MLKL were all lower than that in the Model group (*P* < 0.05), indicating that EA could promote autophagy flux and inhibit necroptosis at the same time. It proved that autophagy may have relationship with necroptosis regulation. Blockage of autophagy flux by CQ may lead to the accumulation of the autophagosomes and increase of P62 expression, which may recruit and interact with RIPK1 to the autopahgosomes and then activate necroptosis. By promoting autophagy flux, Jia-Ji EA could inhibit the necroptosis of neuron, then further inhibit the reduction of the number of anterior motor neurons, and eventually improve the recovery of motor function. Furthermore, we still need to extend the observation time to the regeneration and repair period of SCI in the future study.

## Conclusion

Together our data suggest that, Jia-Ji electro-acupuncture may improve the motor function of hind limbs in rats after SCI by improving lysosomal function, promoting the autophagy flux, then reducing necroptosis and inhibiting the loss of neuron. It showed that the mechanism of Jia-Ji electro-acupuncture treatment were involved in lysosome/autophagy flux/necroptosis pathway. This study provides a potential mechanism and a better understanding of the neuroprotective effect of the electro-acupuncture. In future studies, we will explore the mechanism of Jia-Ji electro-acupuncture on lysosomal function and whether it could induce neuron regeneration and remodeling through this pathway.

## Data Availability Statement

The original contributions presented in the study are included in the article/Supplementary Material, further inquiries can be directed to the corresponding author/s.

## Ethics Statement

The animal study was reviewed and approved by the International Council for Laboratory Animal Science (ICLAS) and Animal Care and Use Committee of China.

## Author Contributions

YH and TH designed and performed most part of the research. LQ performed statistics. FD performed electron microscopy experiment. LG and LX contributed important reagents and provided technical assistance. GF collected, analyzed data, and wrote the manuscript. SZ supervised and assisted the process of work. All authors contributed to the article and approved the submitted version.

## Conflict of Interest

The authors declare that the research was conducted in the absence of any commercial or financial relationships that could be construed as a potential conflict of interest.
