# Supplementary figures and images for: Jia-Ji Electro-Acupuncture Improves Locomotor Function With Spinal Cord Injury by Regulation of Autophagy Flux and Inhibition of Necroptosis
Source: Front Neurosci. 2021 Jan 22;14:616864. doi: 10.3389/fnins.2020.616864 (PMC7862567; doi:10.3389/fnins.2020.616864)

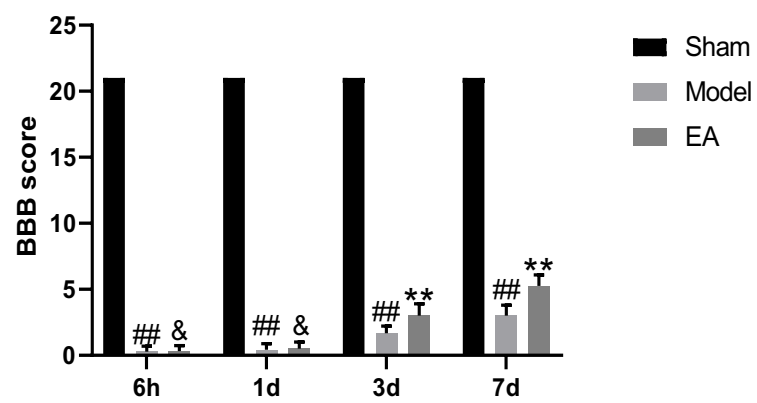

**A**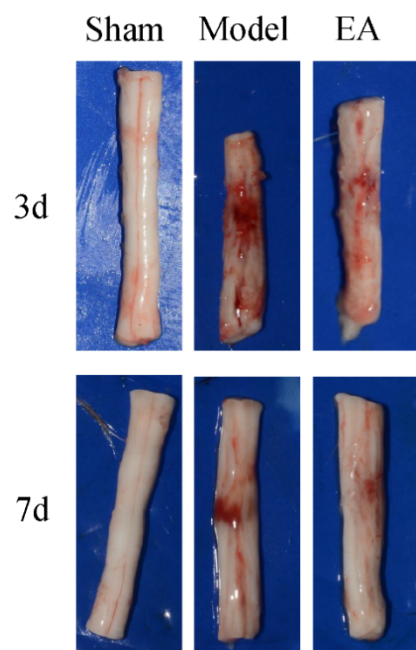**B**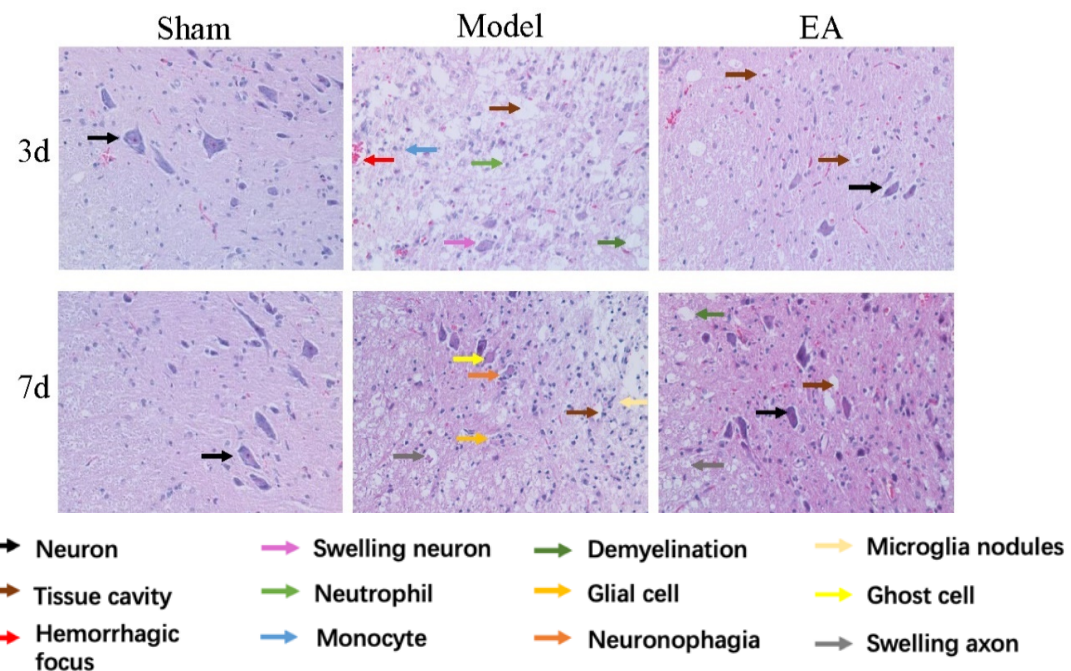**C**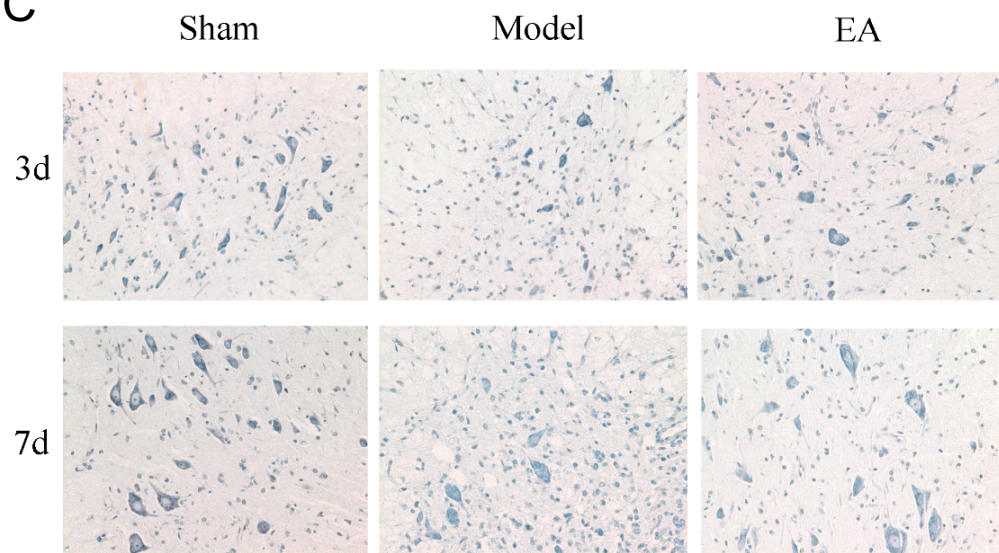**D**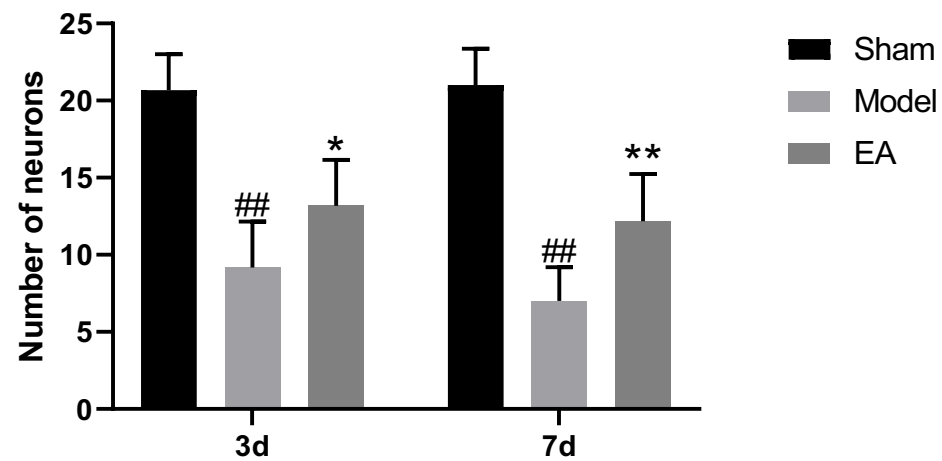

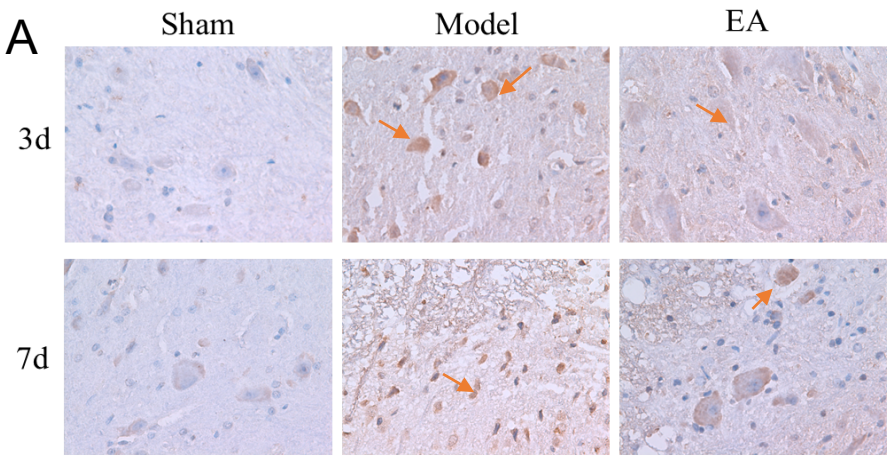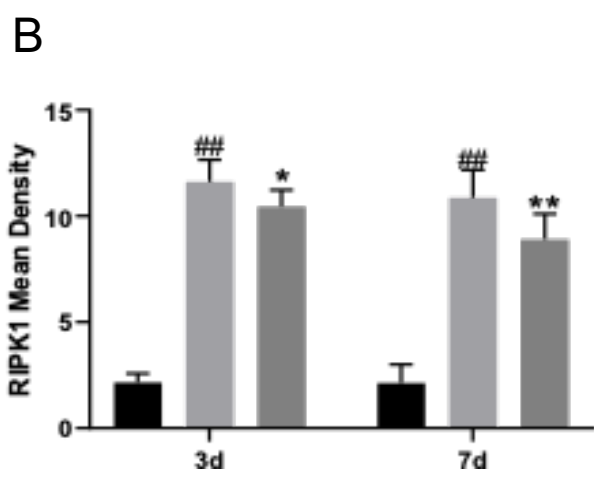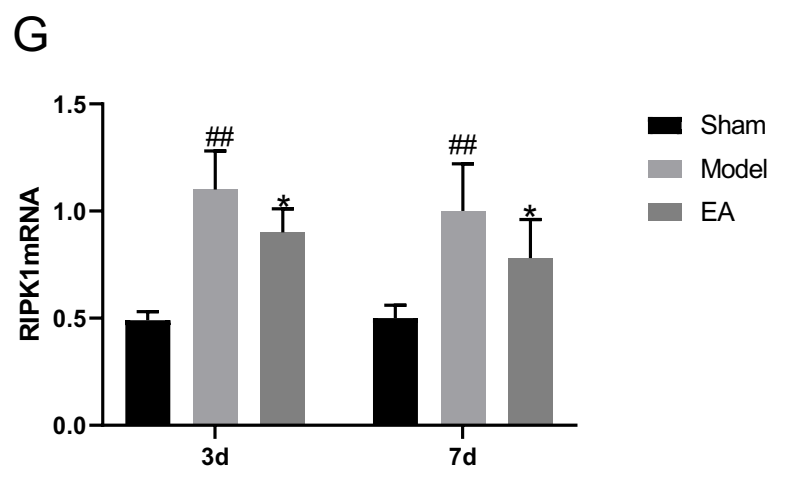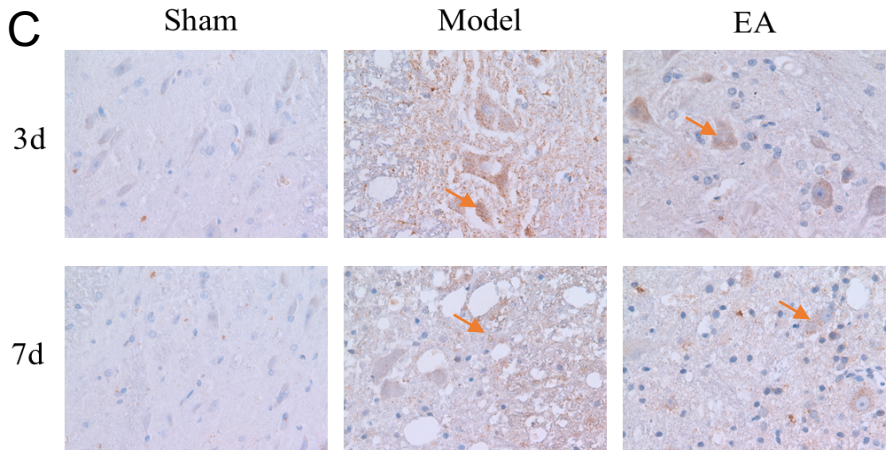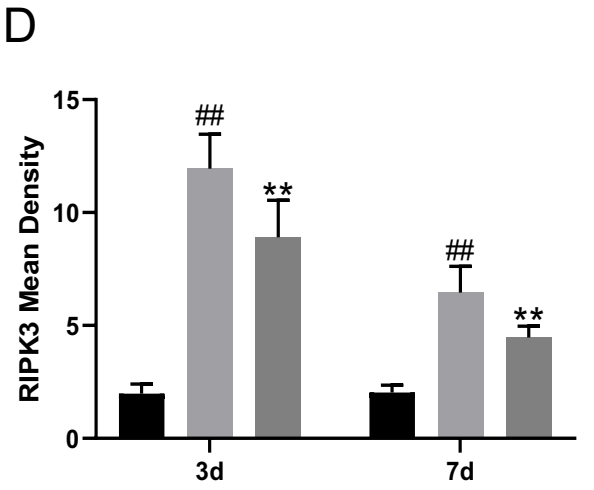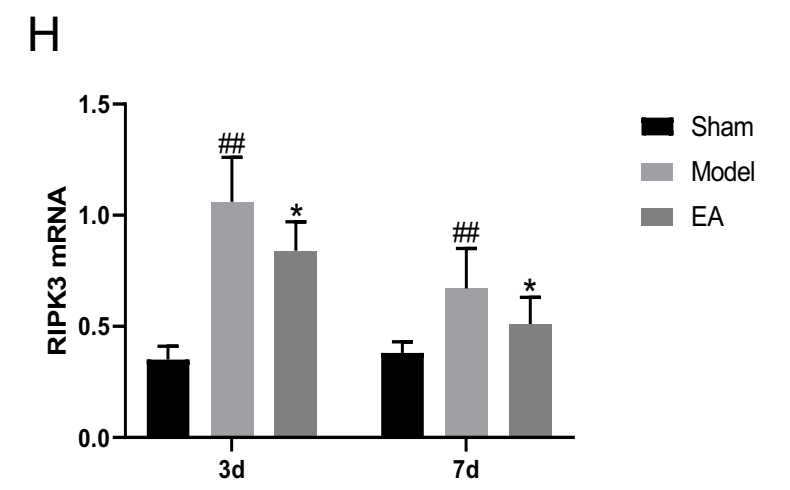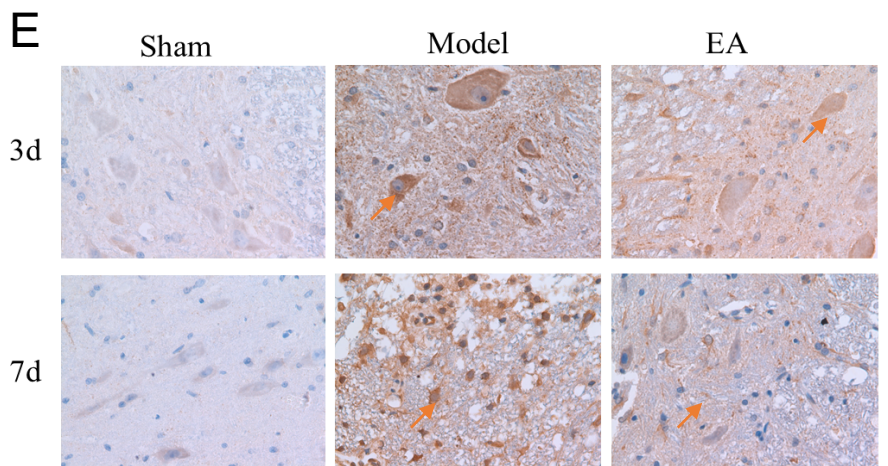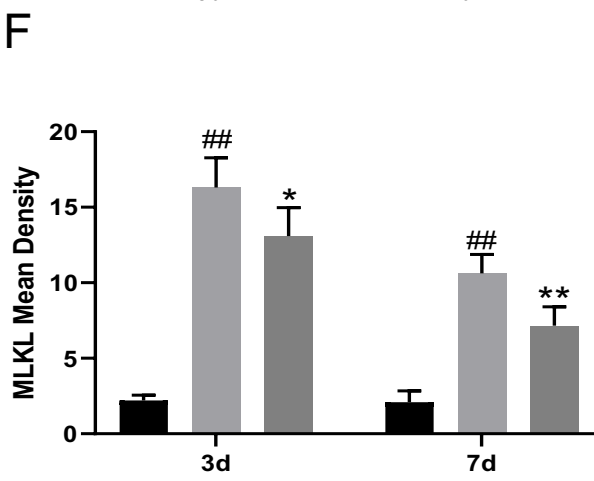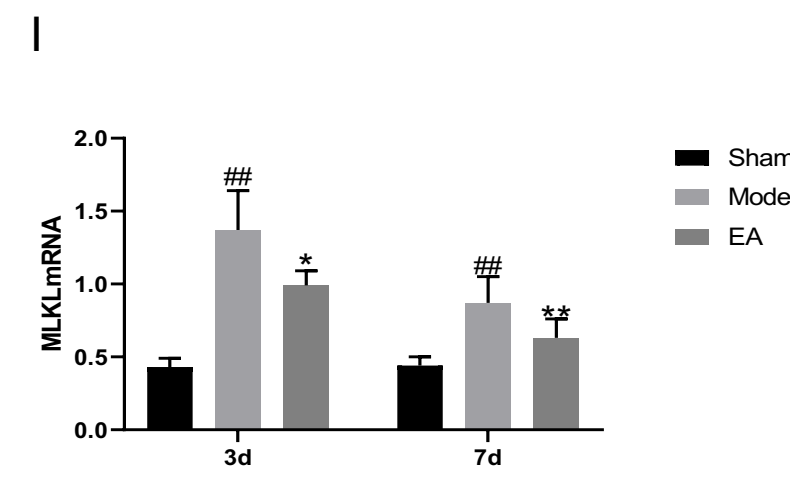

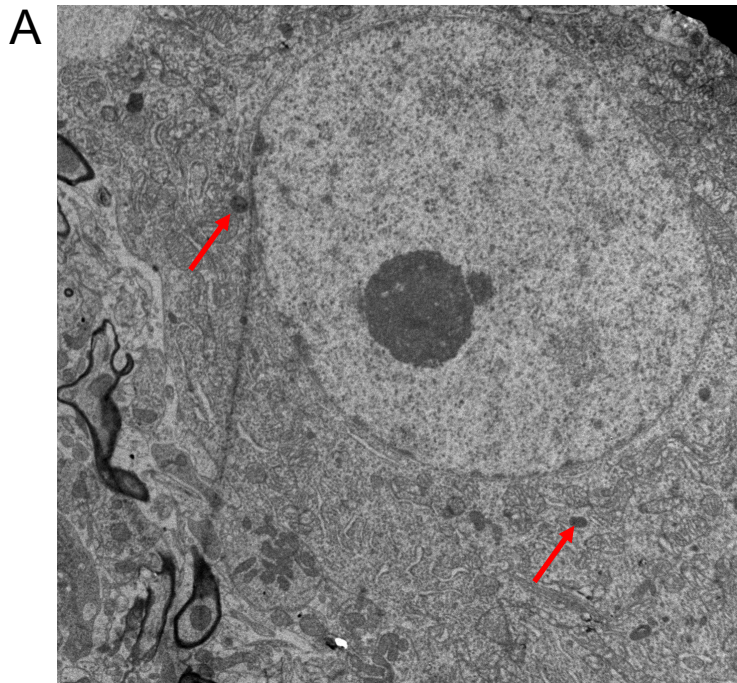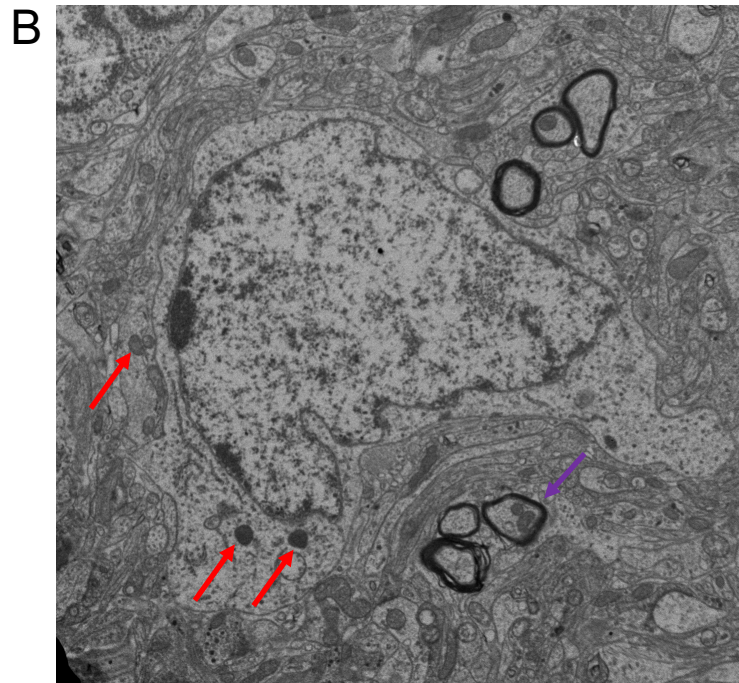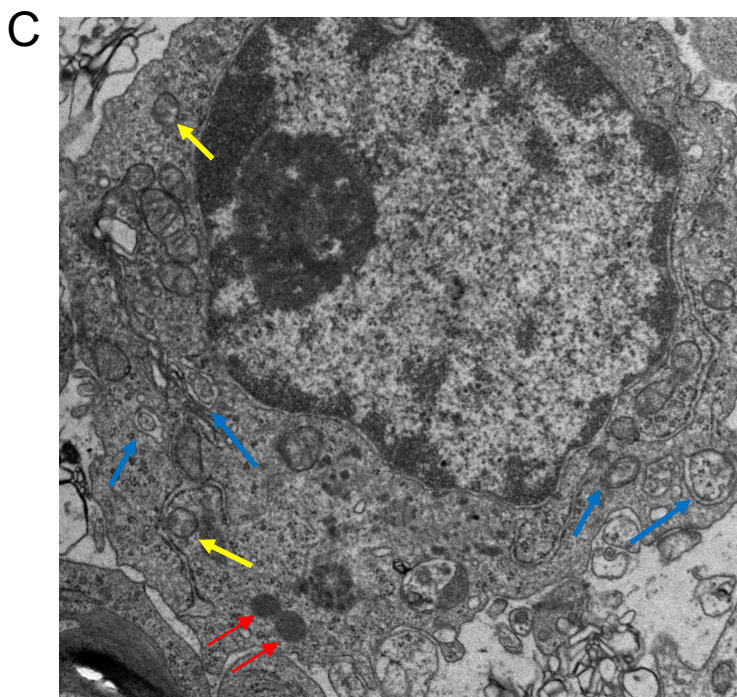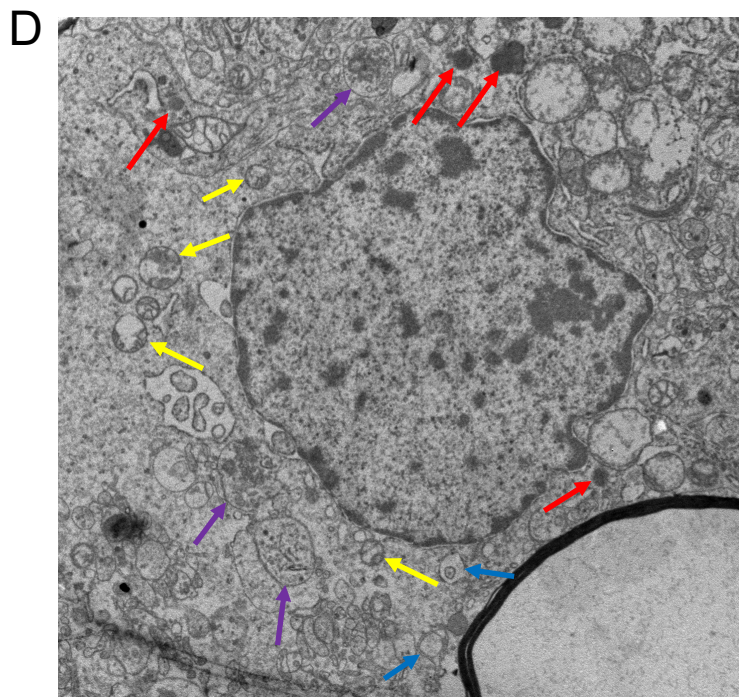

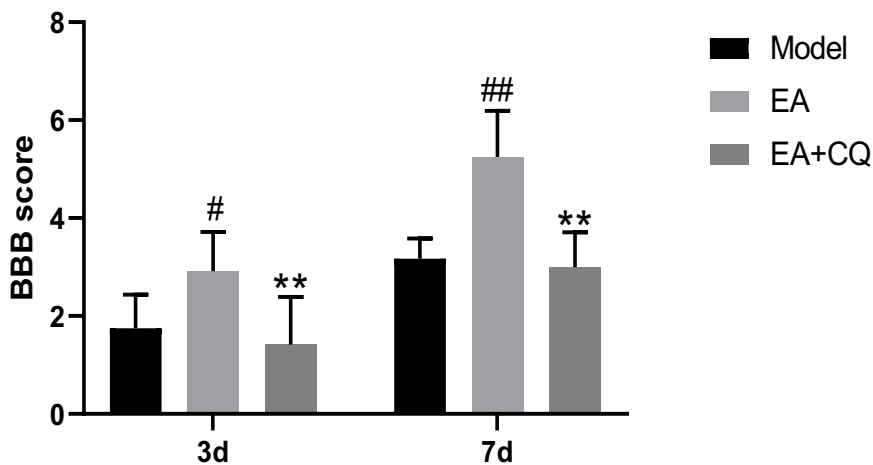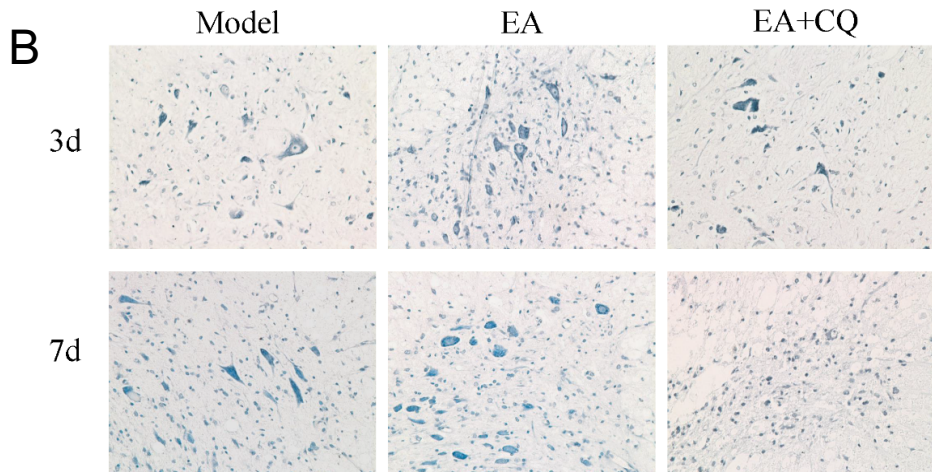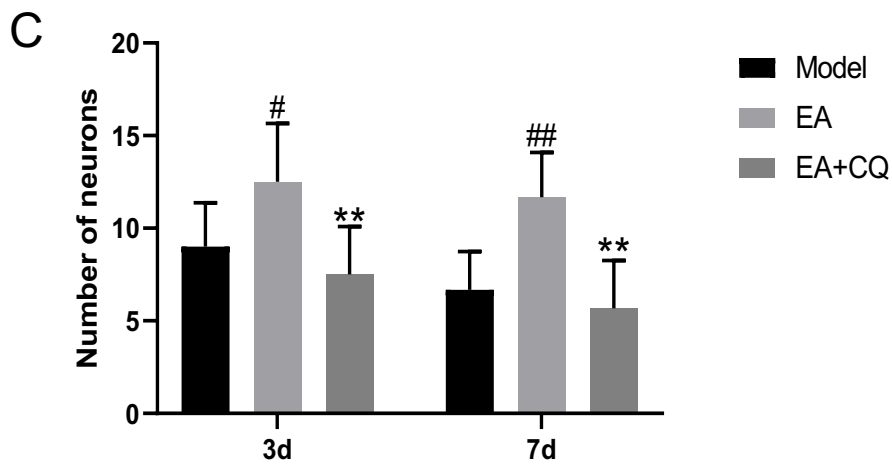

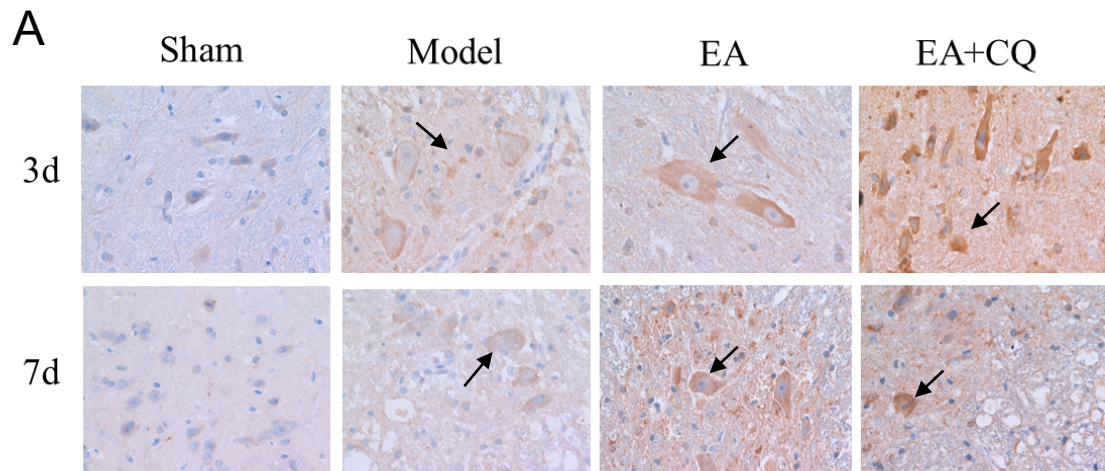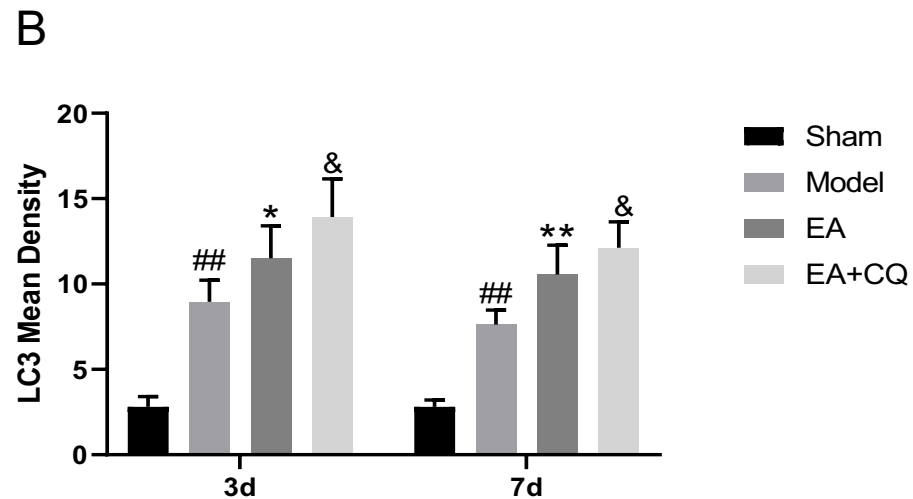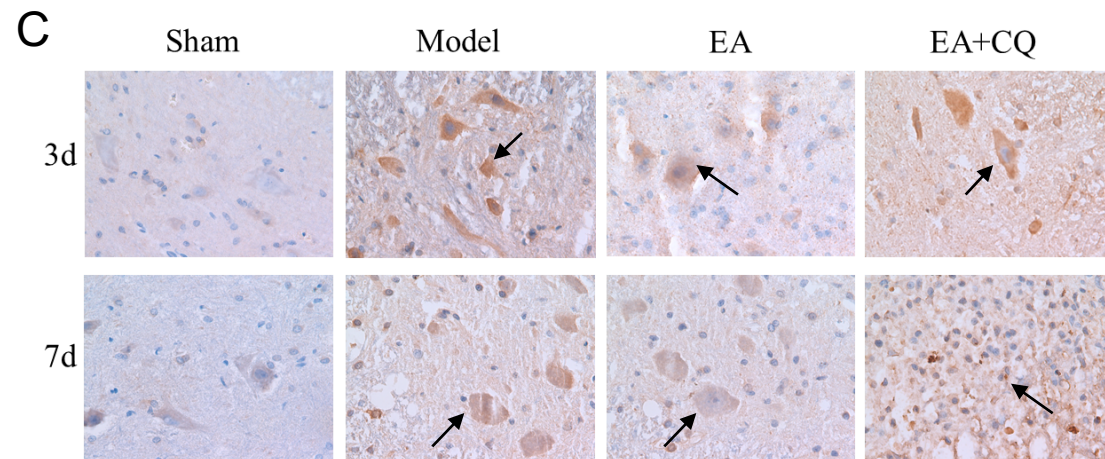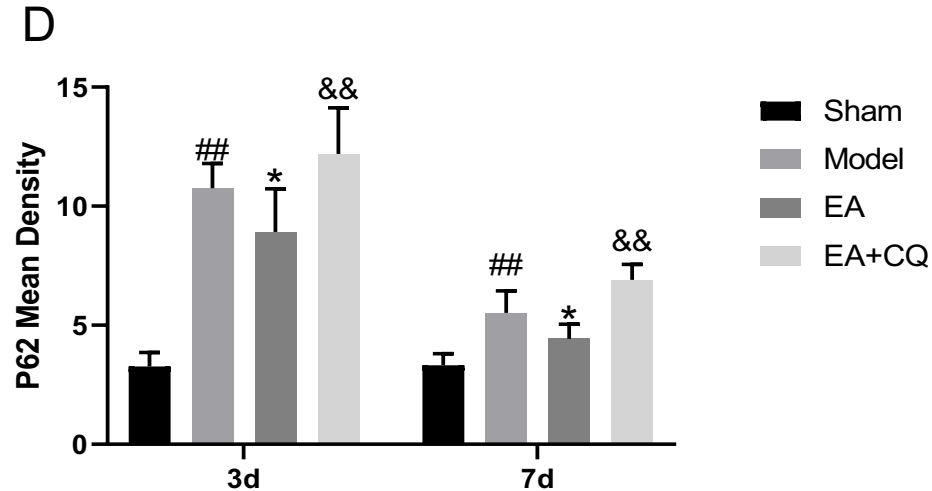

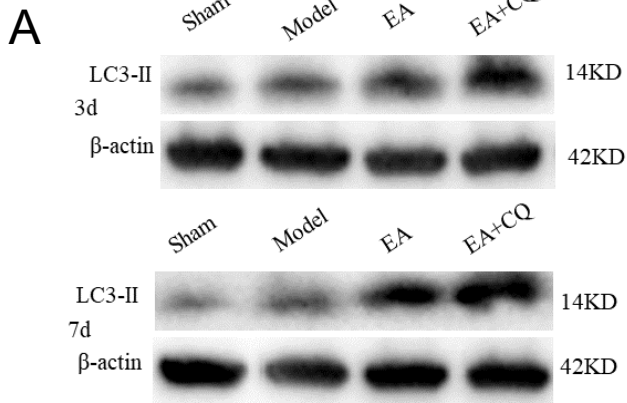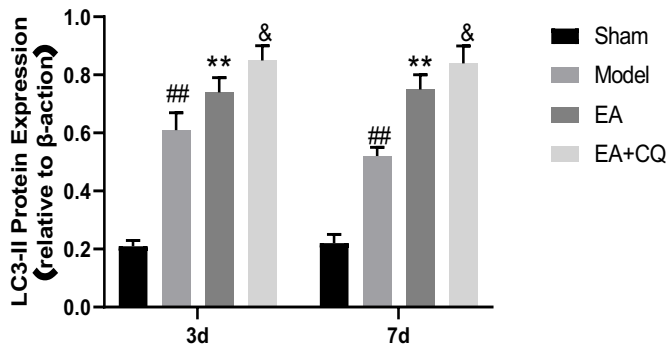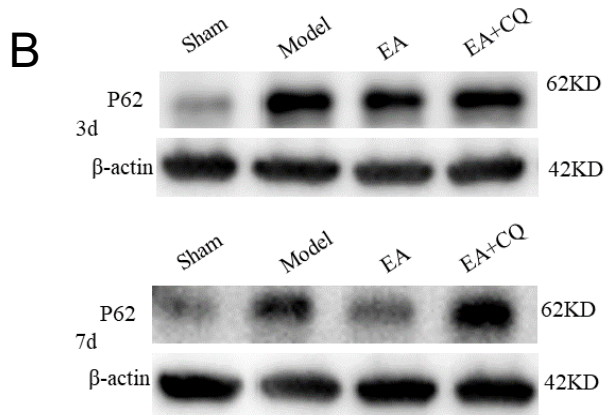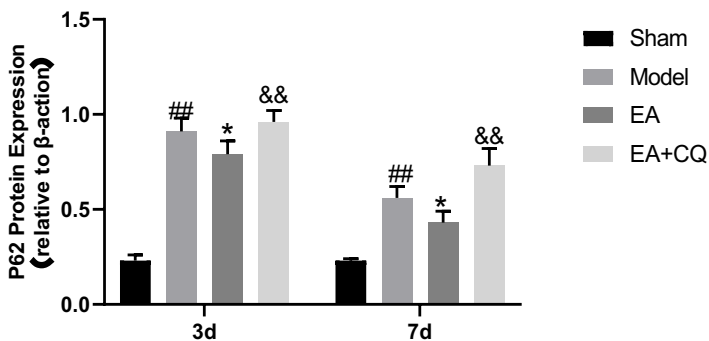

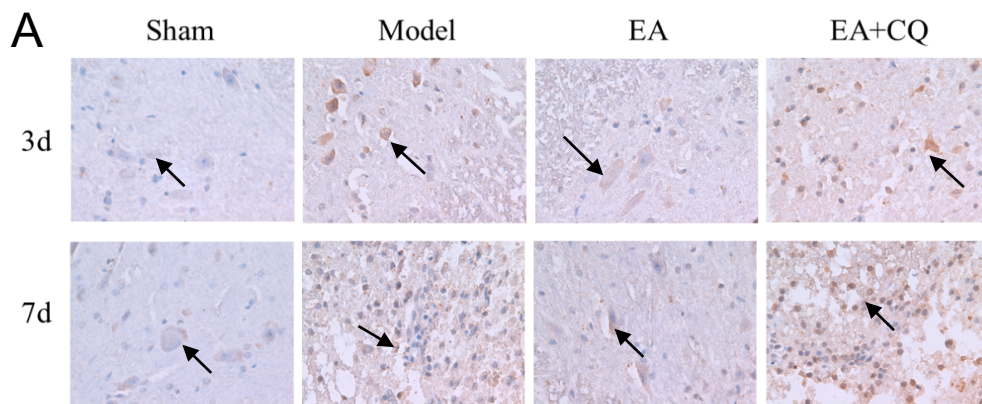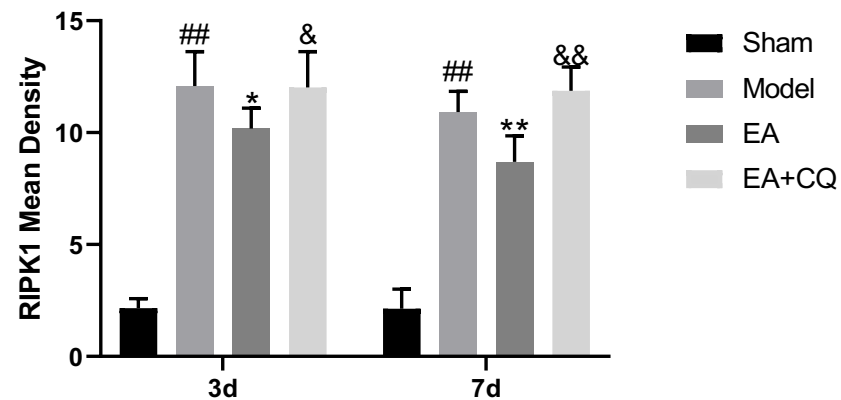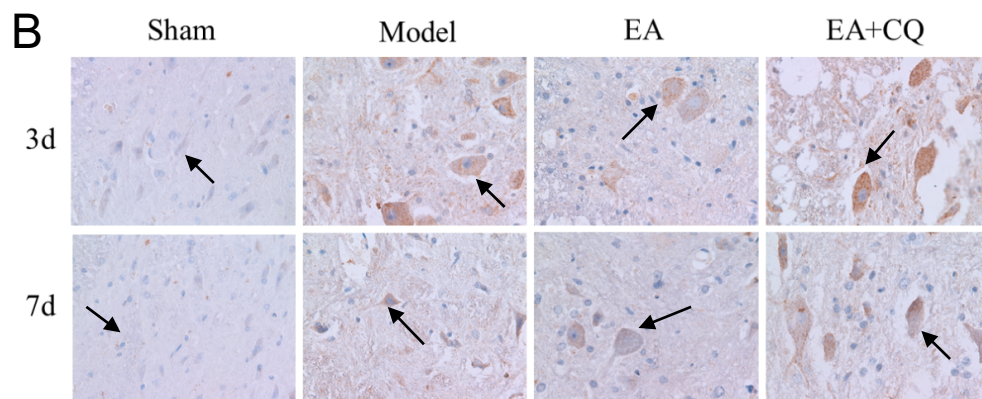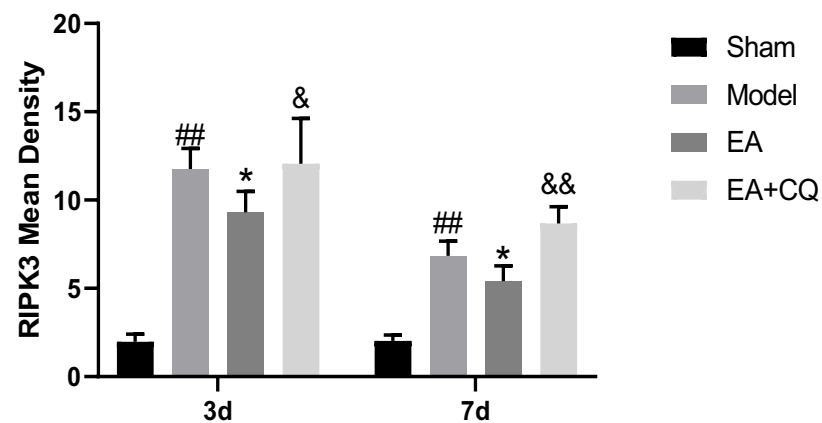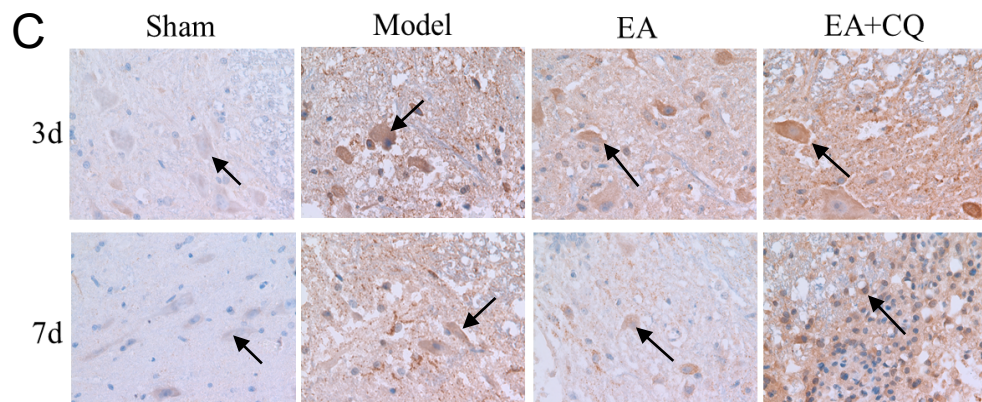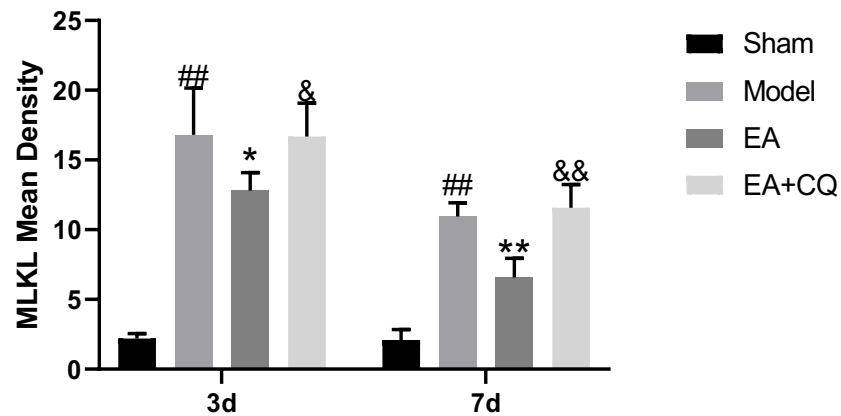

**A**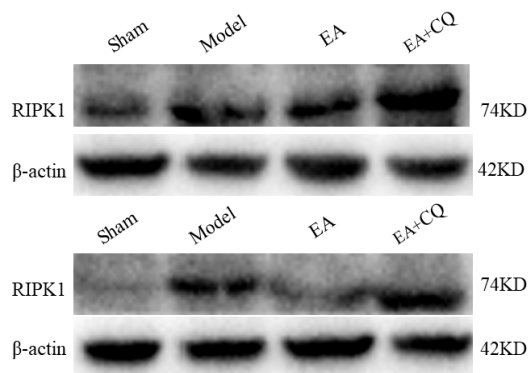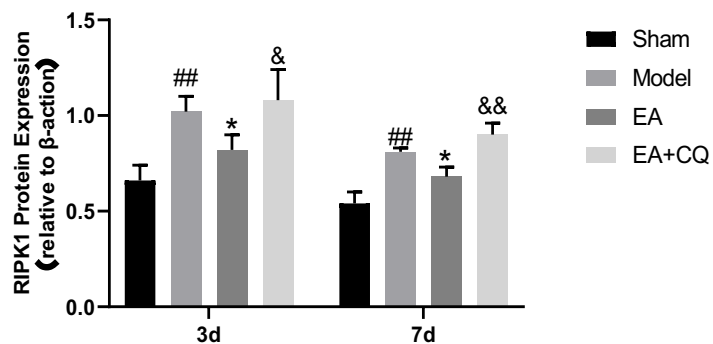**B**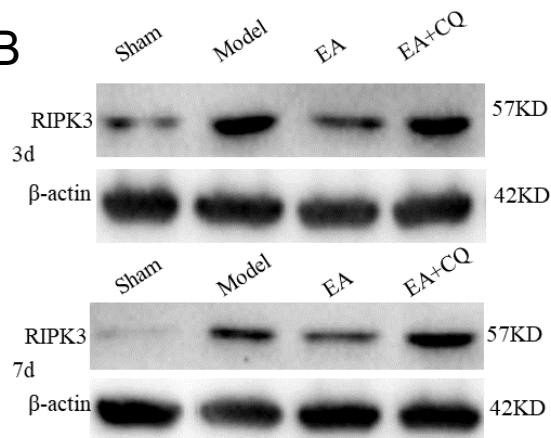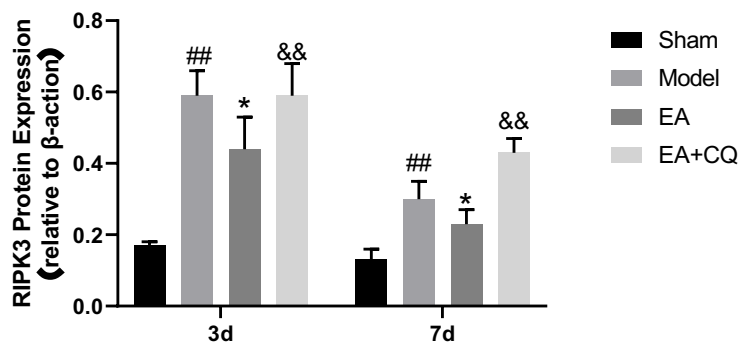**C**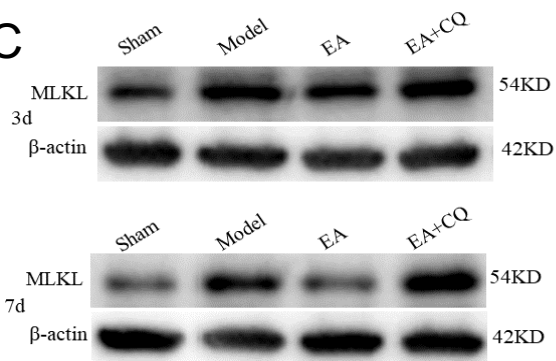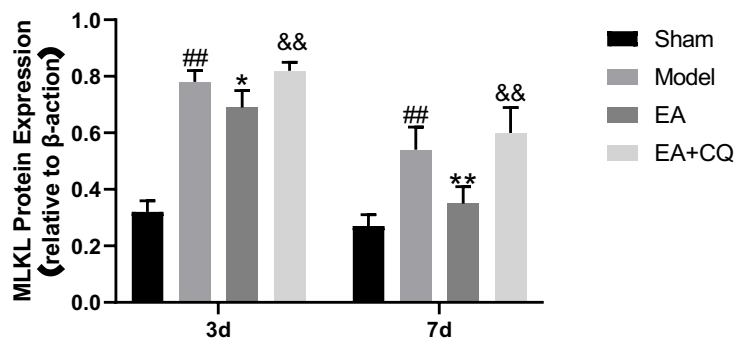

Supplement: Supplementary file 1 [file Data_Sheet_1.PDF]
